# The evolving landscape of minimally invasive procedures in musculoskeletal diseases—part I

**DOI:** 10.3389/fmed.2026.1826391

**Published:** 2026-07-10

**Authors:** Fernando Saraiva, Esperanza Naredo

**Affiliations:** 1 Serviço de Reumatologia e Doenças Ósseas Metabólicas, Unidade Local de Saúde de Santa Maria, Centro Académico de Medicina de Lisboa, Lisbon, Portugal; 2 Department of Rheumatology and Bone and Joint Research Unit, Hospital Universitário Fundación Jiménez Diaz, IIS-FJD, and Universidad Autónoma de Madrid, Madrid, Spain

**Keywords:** ultrasound guidance, minimally invasive musculoskeletal procedures, botulinum toxin, corticosteroids, gene therapy, hyaluronic acid, hydrodissection, platelet-rich plasma

## Abstract

This review aims to cover a wide array of minimally invasive musculoskeletal procedures developed to address several articular and periarticular conditions locally. Besides details related to ultrasound guidance, in part 1, we will review barbotage, botulinum toxin, corticosteroids, dry needling, gene therapy, hyaluronic acid, hydrodissection and mesenchymal stem cells. In part II, we will cover nerve blocks, ozonetherapy, platelet-rich plasma and derivatives, prolotherapy, radiopharmaceuticals, sclerotherapy, thermal ablation and intratissue percutaneous electrolysis. A review of guidelines covering these issues will also be presented.

## Introduction

Rheumatic musculoskeletal diseases (RMD) present significant and persistent challenges to healthcare providers. These conditions can profoundly affect patients’ well-being, functional capacity, and overall quality of life. Furthermore, RMDs often impose a considerable burden on families and society at large, with wide-reaching socio-economic implications and potential consequences for mental health.

There is a growing array of local techniques, specifically minimally invasive musculoskeletal procedures (MIMSP), most of them being ultrasound-guided, developed to address articular and soft tissue manifestations of rheumatic diseases. These interventions are expanding the therapeutic landscape yet simultaneously challenging the existing knowledge and skillset of rheumatologists.

The administration of drugs or autologous products directly into articular or soft tissue sites offers distinct advantages compared to systemic delivery. These include reduced systemic exposure and associated adverse effects, enhanced local bioavailability and, in some cases, lower overall costs. Despite these benefits, the effectiveness of certain intra-articular and soft tissue therapies remains a subject of debate. This uncertainty stems from limited data, methodological shortcomings, study heterogeneity, and a scarcity of high-quality evidence. Additionally, outcomes as reported by patients, such as pain relief and reduction in stiffness, are particularly susceptible to placebo effects, a phenomenon that is especially prominent with injectable treatments.

Conducting rigorous investigations into these therapies is essential, particularly as the demand for effective management strategies continues to rise. Such approaches must be tailored to the unique needs of individual patients, ensuring that treatment remains both patient-centered and clinically relevant.

This review aims to cover the main types of MIMSP in rheumatology, to understand their indications, limitations, and potential, to identify their main adverse events, and to assess the current evidence-based efficacy of MIMSP (1–3). This review emphasizes ultrasound-guided procedures and excludes synovial and muscle biopsies, which are covered elsewhere (4, 5). After the Ultrasound Guidance section, the remaining techniques are depicted in alphabetical order.

## Methods

A literature search was conducted using PubMed and Google Scholar as web search engines. Included key words were “minimally invasive musculoskeletal procedures,” “ultrasound guidance” AND “musculoskeletal local injection,” OR “musculoskeletal injection,” “barbotage,” “percutaneous lavage,” “botulinum toxin” AND “musculoskeletal local injection,” OR “musculoskeletal injection,” “corticosteroids” AND “musculoskeletal local injection,” OR “musculoskeletal injection,” “steroids” AND “musculoskeletal local injection,” OR “musculoskeletal injection,” “glucocorticoids” AND “musculoskeletal local injection,” OR “musculoskeletal injection,” “dry needling” AND “musculoskeletal local injection,” OR “musculoskeletal injection,” “fenestration” AND “musculoskeletal local injection,” OR “musculoskeletal injection,” “percutaneous tenotomy,” “gene therapy “AND “musculoskeletal local injection,” OR “musculoskeletal injection,” “hyaluronic acid” AND “musculoskeletal local injection,” OR “musculoskeletal injection,” “viscosupplementation,” “hydrodissection” AND “musculoskeletal local injection,” OR “musculoskeletal injection,” “brisement,” “mesenchymal stem cells” AND “musculoskeletal local injection,” OR “musculoskeletal injection,” and “mesenchymal stromal cells” AND “musculoskeletal local injection “OR “musculoskeletal injection.” No a priori exclusions were made concerning study design, publication date, or language. The only inscribed restriction was that the search should be limited to human studies. The search produced 2,584 results, of which 2,412 were excluded due to duplications, non-human studies, procedures for non-musculoskeletal applications, or for tumor pathologies. Finally, 172 publications were analyzed, aiming to retrieve information that could be useful for this review.

## Ultrasound Guidance

Before any MIMSP, it is advisable to perform an ultrasound (US) to identify the target and its relationship with the surrounding structures. Additionally, an evaluation with color/power Doppler may be useful to identify vessels that need to be avoided in the nearby area.

We know that quite often US changes the decision to inject and where to inject, and that blind procedures are problematic in small or deep-seated targets. On the other hand, US guidance of MIMSP optimizes fluid detection and aspirated volume, increases efficacy and tolerance of local injectates, reduces adverse events, and is more cost-effective than landmark or fluoroscopic-guided procedures (1, 6–14). This is why MIMSP under US guidance are particularly indicated when non-guided procedures have failed. However, US-guided MIMSP may be more resource-consuming, require additional preparation and training, and in some situations may produce outcomes not meaningfully different from landmark-guided procedures (11, 15). Other advantages of US-guided MIMSP over fluoroscopic guidance, being both techniques available with similar operator expertise, relate to the absence of radiation, the better visualization of soft tissues, and the lower resource consumption with US-guided MIMSP (11). Resource consumption, costs, availability, or radiation are all issues that compare US favorably with CT, MRI, or fusion images with US, in guiding procedures at the peripheral skeleton.

**FIGURE 1:**
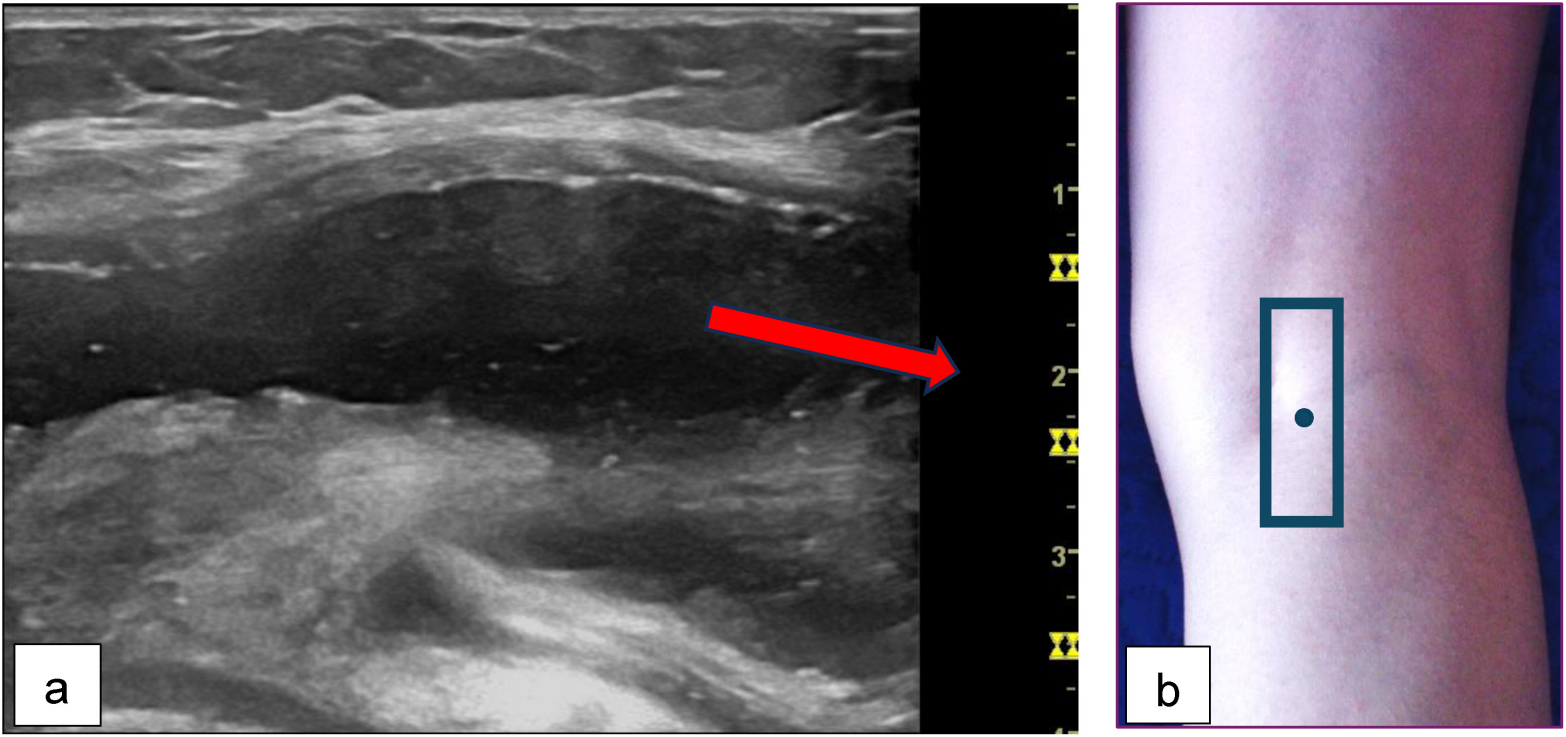
Semi-guided or indirect marking: **(a)** measuring the depth to the target; **(b)** marking on the skin.

US-guided MIMSP may be conducted in two ways—by marking on the skin (semi-guided or indirect), or under direct vision. This one may be performed with free hands or using a guiding device according to the probe’s long or short axis (1). With the indirect option, we use US to depict the target. Then we mark on the skin the area where the probe is, and if we insert the needle in the center of it and penetrate according to the measured depth to the target, we will hit it (Figures 1a,b).

**FIGURE 2:**
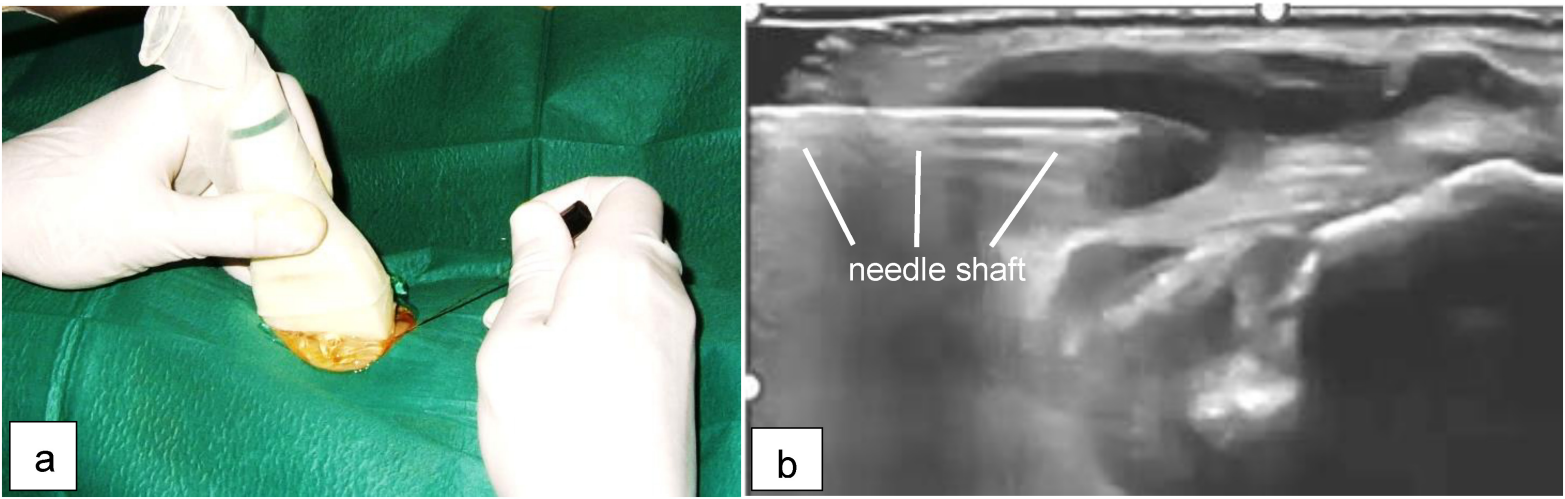
In-plane approach: **(a)** needle and probe position; **(b)** US image.

An easier and preferable way of conducting a US-guided MIMSP is under direct vision. In the in-plane approach, the entire needle is depicted as a hyperechogenic device as it progresses through the tissue until it reaches the target (Figure 2). When performing the in-plane approach, if the operator’s eyes are lined up with the needle shaft, the short axis of the probe, and the US machine, the accuracy of the procedure increases. This is why the most common and preferred modality of US-guided MIMSP is an in-plane free-hand technique, under real-time direct vision.

**FIGURE 3:**
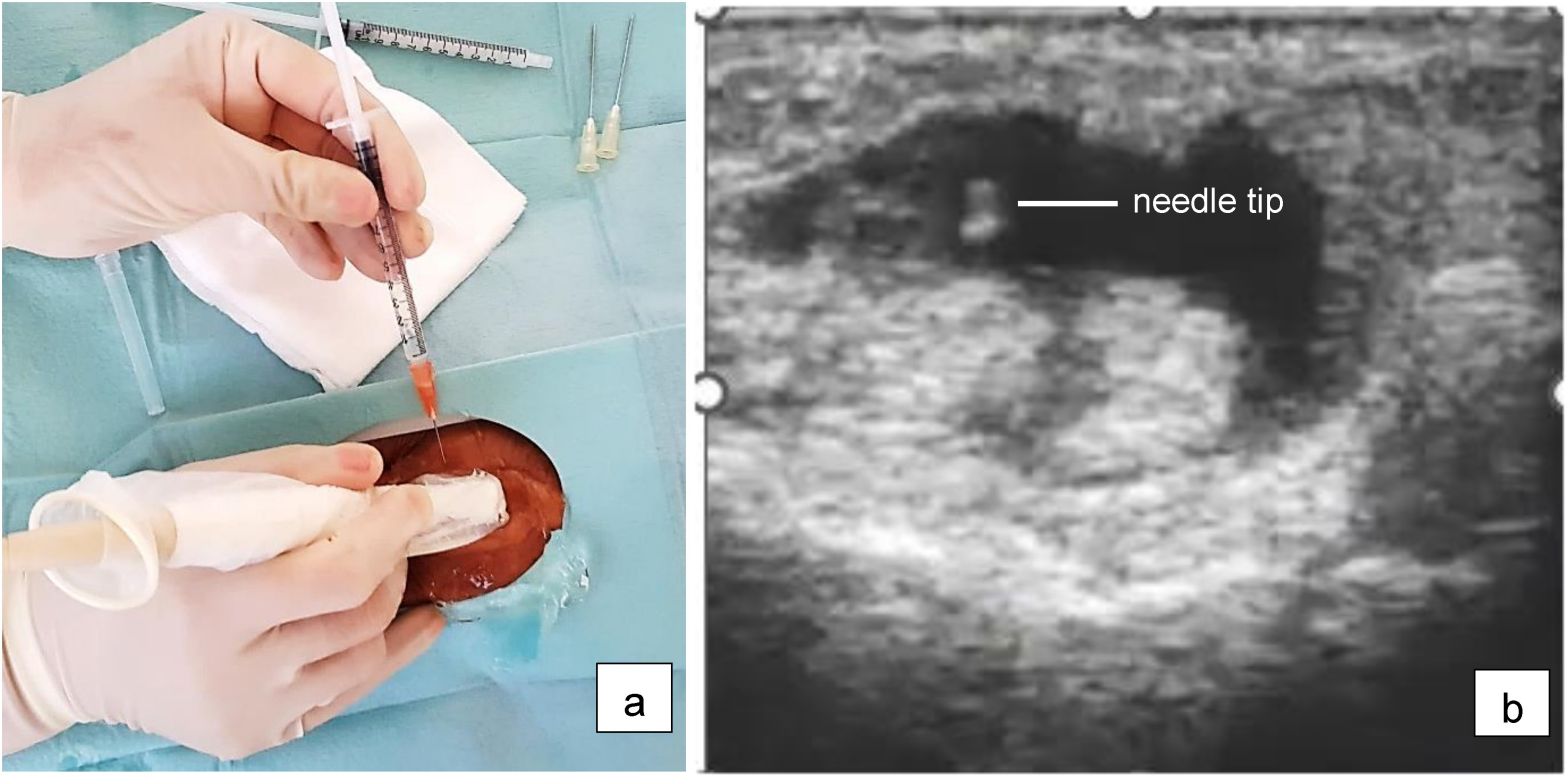
Out-of-plane approach: **(a)** needle and probe position; **(b)** US image.

In the out-of-plane approach, the needle is depicted as a hyperechogenic dot, but only in the moment it intersects the US plane (Figure 3). The major disadvantage in the out-of-plane approach is that the location of the needle tip cannot always be determined.

Careful pre-procedure planning is the most crucial step in successfully performing an ultrasound-guided procedure. Before any US-guided MIMSP, there are a few steps that need to be undertaken, namely (4):

- patient reception and information
- exclusion of contraindications to conducting the procedure
- obtaining written informed consent
- brief ultrasound examination to plan the most adequate needle path
- asepsis and field preparation
- fluid aspiration when present
- local anesthetic when adequate
- the procedure itself

Any US-guided MIMSP must be conducted by qualified operators, with adequate assistance from technical personnel and under adequate aseptic conditions (11, 16).

US-guided MIMSP may be conducted for diagnostic or therapeutic purposes:

- **Diagnostic**
  fluid aspiration
  biopsies
    synovial—joints, bursae, tendon sheaths
    muscle

- **Therapeutic**
  local injections
  other percutaneous needle therapies

**FIGURE 4:**
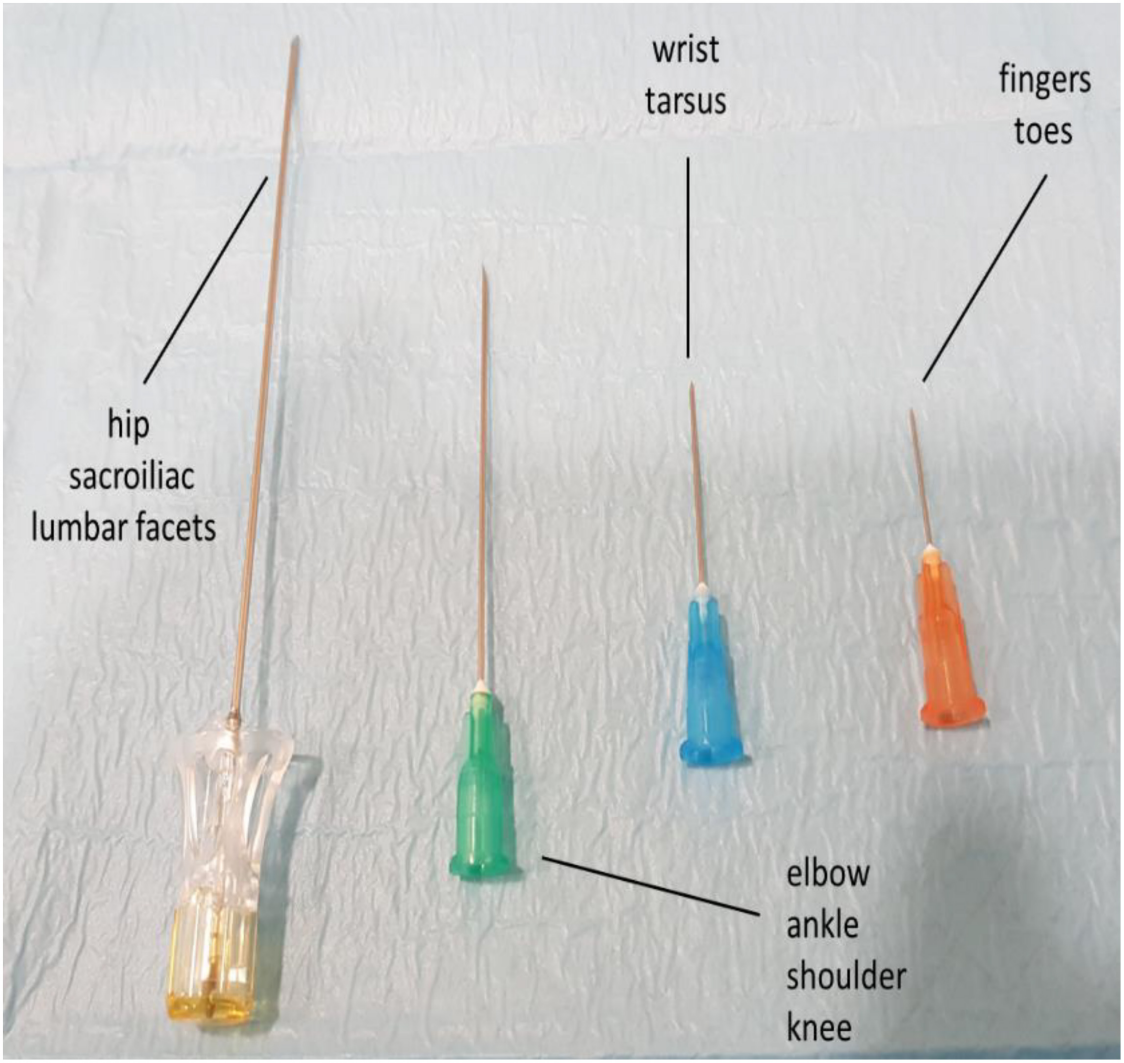
Needle choice according to target.

We need to choose needles according to the size and depth of our targets. In Figure 4, we show examples of this.

Besides synovial fluid, aspirated fluid may also be blood or pus. In every case, its content may be used for biochemical or microbiologic analysis and for cell count, and in the case of synovial fluid, also to search for crystals in the polarized-light microscope (17).

Aspirating fluid may be therapeutic by itself, by removing deleterious cytokines and enzymes, or through structure decompression, and may be conducted for research purposes as well.

Indications for joint and periarticular injections include (2, 3, 18–39):

- **Joint injection**
  synovitis
  osteoarthritis

- **Periarticular injection**
  tendinosis, tenosynovitis
  bursitis and synovial cysts
  fasciitis
  entrapment neuropathies and neuromas
  joint analgesia in severe arthropathies with no indication for joint injection or surgery

Contraindications for MIMSP include skin or systemic infection, coagulation disorder or anticoagulant therapy that might represent an increased risk for bleeding, the uncooperative patient, and allergy to any of the drugs used (17).

General adverse events (AE) of local injections may be occasional, uncommon, or rare (9, 19):

**Occasional**
  vasovagal reaction
• **Uncommon**
  aseptic synovitis
  allergy to the compound
  ecchymosis/hematoma
  nerve lesion
• **Rare**
  skin fistula
  osteonecrosis
  iatrogenic infection
  tendon rupture

The most frequently injected joints are knees, shoulders, wrists, and finger joints, and the most used injectables are corticosteroids, followed by hyaluronic acid (40). Concerning intra-articular therapies, the frequency of AE is low, without clear differences between injectables (19).

Recommending patients to avoid overuse of the injected region, for up to 48 h, may optimize therapeutic results and minimize product linkage, access to vessels, and systemic diffusion.

## The Procedures

### Barbotage/percutaneous lavage

**FIGURE 5:**
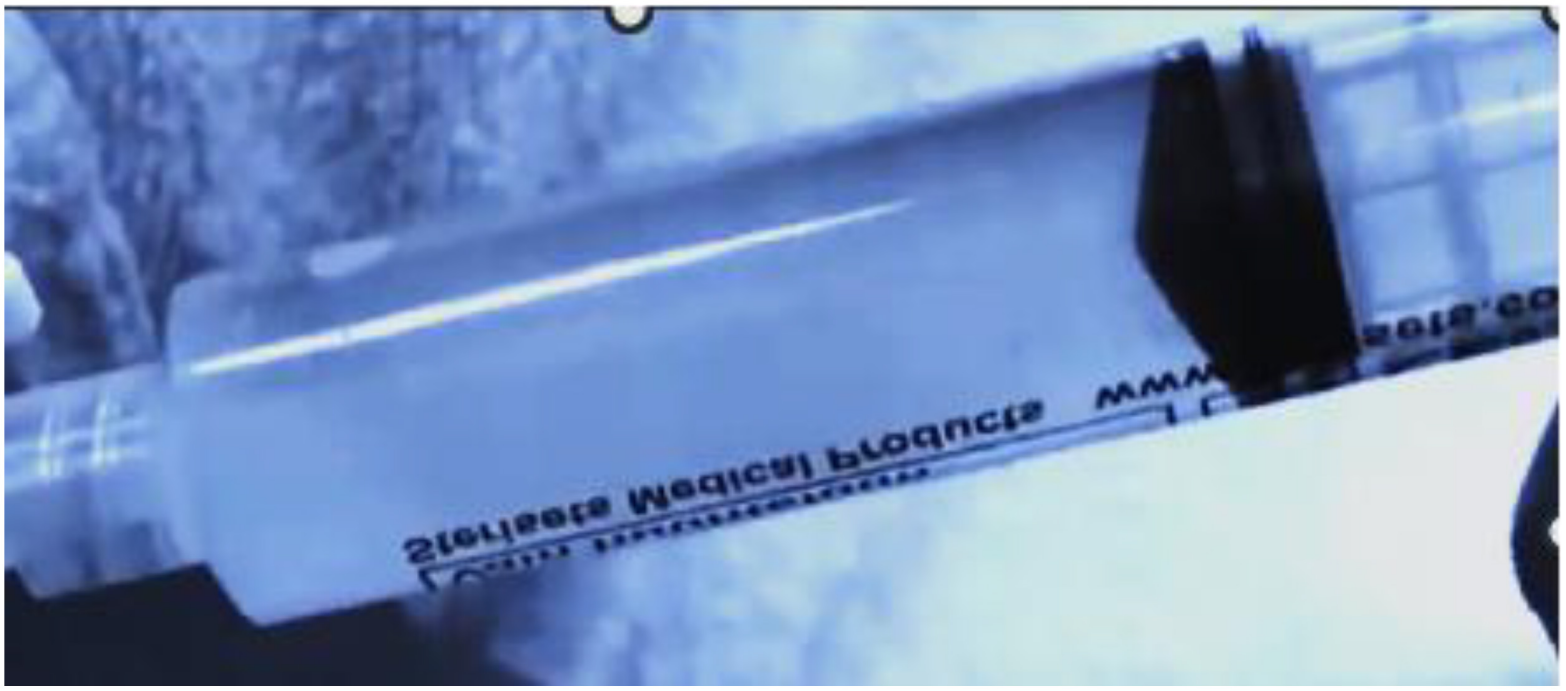
Barbotage with whitish fluid inside the syringe.

The common indication for barbotage or percutaneous lavage is calcific tendinosis of the rotator cuff. It uses a local anesthetic (LA) for tracking the needle up to the calcification and for the subacromial-subdeltoid bursa (SASDB), normal saline (NS) to “wash” the calcification (may be better if warmed up to 37–40 °C), and a corticosteroid (CS), again for injecting the SASDB (41, 42). The syringe that delivers the NS may preferably be of the luer-lock type to prevent spilling of the solution in case of increased injection pressure. A successful procedure is marked by the entry of a whitish fluid inside the syringe (Figure 5).

It can involve either a one-needle or a two-needle approach to break up intratendinous calcifications. The needle should be in close contact with the calcification, and in the two-needle approach, the bevels of the needles should be facing each other, with the injecting needle at the bottom and the aspirating needle on top of the calcification. The one-needle approach may be better for soft calcifications and the two-needle approach for hard ones. However, a systematic literature review showed no evidence favoring the use of a specific size or number of needles, in terms of results or safety (43).

The procedure is not indicated in asymptomatic patients, if the calcification is inside a ruptured tendon, if the calcification is fragmented or smaller than 5 mm, if it has migrated to the SASD bursa, or if it is eroding the bone (41–43).

Specific AE of barbotage include bursitis and adhesive capsulitis.

Extracorporeal shock-wave therapy (ESWT) and barbotage (particularly if combined with corticosteroid subacromial injection) seem to be the best options to treat rotator cuff calcific tendinopathy, with long-lasting pain relief favoring barbotage over other options (44–47).

### Botulinum Toxin

Botulinum toxin (BTX) is produced by Clostridium botulinum, with subtypes A and B approved for human usage.

Common indications for BTX injection include OA (knee, shoulder, ankle), lateral epicondylosis, muscle contractures, fasciitis, neuropathic pain (carpal tunnel syndrome, Morton’s neuroma, complex regional pain syndrome), chronic exertional compartment syndrome, low back pain and myofascial pain syndrome, in a single administration with doses ranging from 20 to 200 U, according to the underlying pathology (48–51). Some randomized controlled trials have shown results favoring BTX in shoulder pain (intra-articular and bursal injections), plantar fasciitis, lateral epicondylosis, and neuropathic pain (48–51). On the other hand, additional systematic literature reviews and meta-analyses have revealed contradictory results or did not show a clear benefit of BTX in myofascial pain syndrome, knee OA, fibromyalgia, neck pain, or piriformis syndrome, with several of these studies showing small numbers and low quality (52, 53). It seems reasonable that BTX may be considered in the management of some of these entities, but only when more conventional therapies have failed (50).

Indications of BTX covering neurological disorders and glandular hyperactivity are also quite common but are outside the scope of this review.

The usual onset of BTX action in musculoskeletal indications varies between 1 and 3 weeks and lasts up to 6 months (48).

BTX induces flaccid paralysis by causing temporary chemodenervation and affects pain neurotransmitters and the autonomic and central nervous systems. By blocking molecules like substance P, calcitonin-gene-related peptide, bradykinin, and glutamate, BTX can decrease the transmission of painful stimuli through inhibition/enhancement of ascending/descending pain pathways, but mainly it causes temporary denervation by preventing the release of acetylcholine into the neuromuscular junction, which is a mechanism of action shared by all BTX serotypes (54). All these effects are mediated at the peripheral nervous system level by disrupting the transfer of pain and surface expression of receptors and by desensitization of nociceptors and blockade of neurotransmitter release. At the central nervous system level, BTX acts through effects on pain processing pathways and possibly in signaling pathways of non-neuronal/supporting cells (54).

Specific AE of BTX include cramping, mild dysphagia, transient weakness, muscle atrophy, and breathing difficulties (48, 50, 52, 53).

### Corticosteroids

CS are the most utilized injectables in rheumatology practice (40). Common indications for its use include synovitis, tenosynovitis, bursitis, periarticular cysts, adhesive capsulitis, entrapment neuropathies, neuromas, fasciitis, tendinopathies, and OA (18, 20, 21, 28, 33, 35, 40, 55). However, considering pain relief and function improvement, CS are usually only effective in the short and mid-term, not in the long term (33, 56).

Several CS may be used for intra-articular or peri-articular injection, differing in their relative potency and length of action (Table 1).

**TABLE 1:** Injectable corticosteroids: equivalencies and duration of action.

Corticosteroid	Equivalent dose (mg)	Common brand names	Duration of action (h)
Hydrocortisone	20	Cortef, Solu-Cortef	8–12
Prednisone	5	Deltasone	12–36
Prednisolone	5	Orapred, Prelone	12–36
Triamcinolone acetonide	4	Kenaiog, Kenacort	12–36
Triamcinol. hexacetonide	4	Aristospan, Lederspan	24–36
Methylprednisolone	4	Depo-Medrol, Solu-Medrol	12–36
Dexamethasone	0.75	Decadron	36–54
Betamethasone	0.6	Cel estone	36–54

Concerning osteoarthritis, positive results have been shown for intra-articular glucocorticoid injection in the knee, hip, trapeziometacarpal, and acromioclavicular joints (57–62). Intra-articular CS also have a recognized benefit in rheumatoid arthritis, juvenile idiopathic arthritis and spondylarthritis (psoriatic arthritis included), notably in hand metacarpophalangeal and proximal interphalangeal joints, wrists, knees, metatarsophalangeal, ankles, shoulders and elbows, but only in the short-term, as well (63–65). Local CS injections are the treatment of choice for inflammatory tenosynovitis and bursitis, for trigger-finger and De Quervain’s disease (66). For other tendinopathies, local CS also show positive results, but there are concerns regarding tendon damage after repeated injections, and CS are not indicated if there is a pre-existing tendon rupture or in Achilles or patellar tendinopathies, in which they do not show any benefit (33). For lateral epicondylosis, CS injections produce doubtful outcomes, and their use will depend on the availability of local alternatives.

CS act by blocking the expression of proinflammatory genes, cells, cytokines, and mediators and downregulate immune function (30).

Intra-articular glucocorticoid injections are overall safe, with a reported incidence of adverse events similar to placebo, mostly mild and self-limited (19, 58, 66–69). Overall, the most common AE are post-procedure flushing and vasovagal reaction, in up to 40 and 20% of cases, respectively, and the most common local AE is post-injection flare, which may occur in up to 25% of cases (69).

Other specific short-lived systemic AE include hyperglycemia, which is frequent but only in diabetic patients, lasting 1–3 days, headache (in up to 18% of cases), increased appetite, mood changes and rarely or very rarely adrenal suppression, Tachyon syndrome (dorsal, lumbar or chest pain, associated to restlessness, nausea, skin flushing, sweating or dyspnea), or anaphylactic shock (66, 69, 70).

Other specific short-term specific local AE include tendon rupture (occurs in <1% of CS injections and is more frequent in soft tissue injections), skin hypopigmentation and atrophy/necrosis of subcutaneous tissue (in up to 7% of cases), injection site pain (in up to 6.8% of cases), relevant bleeding in patients under antithrombotic drugs (in up to 0.2% of cases) and rarely or very rarely Nicolau’s syndrome (livedoid lesions and necrotic ulcers in result of micro-emboli obstructing dermal arteries), local calcification or joint-skin fistula (66, 69, 71, 72).

Mid and long-term AE include chondrolysis in repeated intra-articular injections of the knee, rapidly progressive hip osteoarthritis, aseptic osteonecrosis, and Charcot-like arthropathy, which are all rare (57, 66, 73–75).

Septic arthritis is a feared but rare complication of intra-articular CS injection, affecting no more than 0.036% of all cases (16, 69). It is more common (up to 0.08% of cases) in males, advanced age, and in patients with pre-existing joint disease or comorbidities (76).

### Dry needling/fenestration/percutaneous tenotomy

Dry needling (DN), fenestration, or percutaneous tenotomy are different designations that are interchangeably used for a procedure in which there is penetration of a tendon or trigger-point without injecting anything. However, fenestration or percutaneous tenotomy involves gently puncturing the abnormal structure multiple times (15–25 passes), passing a needle through it manually, or using an electronic device. In the case of fenestration, beveled cutting-edge needles or an electrical scalpel are used. However, other modalities of DN exist, including the insertion of a needle once and leaving it in place, for a variable amount of time (acupuncture, if the needle penetrates an acupuncture point), or passing an electrical current through it. Percutaneous electrical nerve stimulation uses a pulsed electrical current, and the needle may be inserted inside the structure to be treated or inside acupuncture points (electroacupuncture). In percutaneous electrolysis, a continuous (not pulsed) electrical current passes through the needle. All these modalities in which the needle is left in place without fenestration use solid filament needles. This is not the case in percutaneous tenotomy, in which a beveled hollow needle is used (2, 77, 78).

DN disrupts abnormal tissue, causing bleeding and releasing cytokines and other proinflammatory mediators, aiming to stimulate a local healing response through granulation tissue formation, cell proliferation, and increased matrix synthesis, leading to tendon reinforcement. Moreover, DN may reduce peripheral and central nervous system sensitization by modulating dorsal horn activity and stimulating descending inhibitory pathways (2, 77, 79).

Common indications of DN include tendinopathies and enthesopathies (Achilles, patellar, hip adductor, rotator cuff, tricipital, and trochanteric tendinopathies/enthesopathies, fasciitis, and medial and lateral epicondylosis) and myofascial pain syndrome. For most of these indications, low-quality evidence points to a beneficial effect of DN. However, high-quality studies are needed to better clarify DN outcomes regarding pain and function improvement, namely not using DN combined, but compared with other treatment modalities and appropriate sham interventions, testing different DN protocols and using ultrasound-guidance (2, 77–91). Considering DN modalities that use electrical currents, issues that need to be addressed relate to treatment duration, intensity of the current, and number of sessions (78).

Common and minor AE of DN include local soreness and bruising, but they are usually time-limited. Major but rare AE include infection, prolonged symptom exacerbation, permanent nerve or tendon lesion, and pneumothorax (78, 92).

DN may or may not be associated with autologous products like autologous whole blood or platelet-rich plasma. This combined approach needs further exploration because there seems to be a trend favoring the association over any of the modalities used alone (81, 88–90, 93).

### Gene therapy

Gene therapy (GT) is an experimental approach that uses specific DNA or RNA sequences to modify gene expression to treat or prevent disease. In musculoskeletal disorders, intra-articular gene delivery aims to introduce complementary DNA (cDNA) or RNA so that therapeutic products can be synthesized endogenously within the joint over a sustained period. Despite this promise, GT for osteoarthritis (OA) and rheumatoid arthritis (RA) remains challenging because both are polygenic conditions without a single clear genetic target. In addition, GT is associated with high development and manufacturing costs, important safety concerns, and a limited evidence base in humans, with most data still derived from animal and preclinical studies (94–99).

Delivered genes may encode growth factors, transcription factors, anti-inflammatory cytokines, signaling molecules, matrix proteins, or receptors. In musculoskeletal disease, these strategies are being explored mainly in OA, tendinopathy, and RA (97).

A major limitation of GT is the transient expression of the gene product, regardless of the vector used. Even so, compared with recombinant protein replacement therapy, gene-based approaches can provide longer-lasting, targeted, and site-specific protein expression more physiologically. GT has also been associated with fewer immune reactions than allogenic stromal cell therapy in some settings (99, 100).

#### Delivery methods

Gene transfer may be performed by either a direct (*in vivo*) or indirect (*ex vivo*) approach. In direct delivery, the vector and transgene are injected locally into the joint. In indirect delivery, healthy cells are first harvested from the patient, genetically modified and expanded in culture, and then re-implanted into the affected joint by intra-articular injection or arthrotomy, with or without a scaffold. Overall, indirect (*ex vivo*) delivery is considered more efficient than direct delivery and safer than introducing viral vectors directly into the body (97, 101).

Injection-based delivery mainly places the therapeutic product in contact with the synovial membrane rather than directly with a chondral or osteochondral defect. For this reason, it is used primarily to deliver anti-inflammatory cytokines such as IL-10 or IL-1ra in RA or in osteoarthritic joints with full-thickness defects. In direct procedures, the vectors most commonly used are recombinant adeno-associated virus (rAAV) and self-complementary adeno-associated virus (scAAV), followed by plasmids and lentiviruses (97).

For cartilage defects, indirect procedures are used more often, with cell vehicles such as mesenchymal stroma cells (MSCs), chondrocytes, or fibroblasts. Vectors may also be incorporated into scaffolds to reduce dilution of modified cells after intra-articular injection (97).

#### Gene delivery vectors

Vectors used in GT may be broadly classified as non-viral or viral. Non-viral vectors are generally safer, easier to handle, less immunogenic, and potentially more cost-effective than viral vectors. However, they usually produce lower and shorter-lived transgene expression, are often limited to the synovial membrane, and have lower transfection efficiency (approximately 25-50%, compared with 70–95% for viral vectors), with poorer targeting than viral vectors (Table 2) (97, 100–102).

**TABLE 2:** Comparison between viral and non-viral vectors.

Feature	Non-viral vectors	Viral vectors
Transfection efficiency	Lower (approximately 25–50%)	Higher (approximately 70–95%)
Targeting	Poorer targeting; often limited mainly to the synovial membrane	Better targeting and broader cell transduction
Safety	Generally safer and easier to handle	Concerns regarding persistence in the host, inflammation, potential spread to other organs, and carcinogenesis
Immunogenicity	Less immunogenic	More immunogenic, although AAV is less immunogenic than other viral vectors
Cost	Potentially more cost-effective	Typically, more expensive to produce
Main advantage	Safety, simplicity, and easier handling	Higher efficiency, longer-lasting and stronger gene delivery performance

Gene delivery vectors include:

**Non-viral vectors**
  lipid-based systems
  liposomes
  polymers
  nucleic acid conjugates
  naked DNA
• **Viral vectors**
  adenovirus
  AAV (scAAV, rAAV)
  retrovirus
  lentivirus
  baculovirus

The main advantage of viral vectors is high transfection efficiency. However, major concerns include persistence of viral material in the host, local inflammation, possible spread to other organs, carcinogenesis, immunogenicity, and high production costs (Table 2) (101).

Adenoviruses are commonly used because they achieve high transduction efficiency and transgene expression in several cell types, making them suitable for *in vivo* approaches and for the transfer of growth factor genes. Their main limitation is the short duration of expression, typically only 1–2 weeks, because the vector remains episomal. Clinical concerns also include humoral and cellular immune responses to adenoviral gene products. To reduce these risks, modified “gutless” adenoviral vectors containing only the packaging sequence and terminal repeats may be used; these offer improved safety but can still trigger innate immune responses if given systemically and require a helper virus for transduction, which adds complexity (95).

Among viral vectors, adeno-associated virus (AAV) is widely regarded as the leading candidate for *in vivo* intra-articular delivery of therapeutic cDNAs. AAV can penetrate deep into articular cartilage, transduce chondrocytes *in situ*, and may support transgene expression for months to years, with some reports suggesting persistence of action for up to 10 years, potentially reducing the need for repeated injections (103). AAV is considered effective and relatively safe, as it is not known to cause disease and is generally less immunogenic than other viral vectors, although immune reactions may still occur after repeated administration (94, 95, 98, 103). The most commonly used serotypes are 2 and 5, and the main forms are recombinant AAV (rAAV) and self-complementary AAV (scAAV). rAAV is derived from the non-pathogenic parvovirus AAV after removal of viral coding sequences, which helps minimize host immune responses. In addition, rAAV does not require cell division or vector integration for gene expression and can sustain high-efficiency expression for extended periods. scAAV has been used for the delivery of IL1-Ra (96, 97, 99, 101). Because AAV cannot replicate independently, it requires complementation by proteins supplied by an adenovirus. Even so, AAV remains the preferred viral vector for *in vivo* delivery, whereas retroviruses are generally preferred for *ex vivo* delivery in both OA and RA (95, 99).

Retroviruses are limited mainly by insertional mutagenesis and the potential activation of oncogenes. For this reason, they are usually used in *ex vivo* settings after irradiation with gamma or X-rays to render viral replication incompetent (97, 101). Lentiviruses, a subclass of retroviruses derived from HIV, can transduce non-dividing cells and have therefore been proposed as vectors. However, concerns remain about introducing HIV-derived genetic material *in vivo* and about vector integration, which may lead to insertional transformation and tumor formation. Although non-integrating lentiviral systems have been developed, they are not yet widely available (97, 98).

Baculovirus is used less often because it neither replicates nor integrates its DNA, resulting in only very transient gene expression, typically for less than 7 days (97).

In cell-based applications, some studies suggest that MSCs have limited chondrogenic differentiation and immunomodulatory capacity even after enhancement with viral or non-viral vectors. In addition, bone formation and fibrocartilage may develop instead of hyaline cartilage (97, 99–101).

In animal models of tendinopathy, exosomes—nanometre-sized extracellular vesicles involved in cell-to-cell communication—have been studied in adipose-derived stem cells, educated macrophages, tendon stem cells, and bone marrow-derived MSCs, with encouraging effects on tendon repair (100).

Non-viral vectors are generally preferred when safety and simplicity are the priority. Viral vectors are generally preferred when high-efficiency gene delivery is needed. AAV stands out among viral vectors because it offers a relatively favorable balance between efficacy and safety. However, only a small number of the approaches described above have begun to reach experimental human intra-articular application, usually in combination with viral vectors (98). One example is a mixture of non-transformed and retrovirally transduced, irradiated allogenic primary chondrocytes overexpressing TGF-β for OA (96, 99, 101, 104, 105).

Overall, intra-articular GT remains at an early stage compared with gene therapy for monogenic diseases, largely because the underlying disorders have complex and still incompletely understood aetiologies (95). Current barriers include transient transgene expression, suboptimal concentrations of target proteins (despite occasionally reaching supra-therapeutic levels shortly after treatment), inability to restore normal cartilaginous architecture, and high cost. Potential strategies to address these limitations include binding motifs that prolong local protein residence time and scaffold-based cell therapies. Nevertheless, important safety questions remain, and robust efficacy data are still required before GT can be adopted routinely in clinical practice (97, 99).

#### Key take-home messages

- Gene therapy remains largely experimental in musculoskeletal medicine, with most evidence derived from preclinical studies.
- AAV vectors currently represent the leading platform because of prolonged expression, cartilage penetration, and a favorable safety profile.
- Major therapeutic targets include anti-inflammatory cytokines, growth factors, transcription factors, and non-coding RNAs.
- Improvements in vector design have improved safety; however, concerns about immune reactions and oncogenesis persist.
- Experimental models suggest enhanced repair with cell-based therapies, although differentiation remains variable and tissue quality is inconsistent.
- Clinical translation is limited by high costs, safety concerns, transient or suboptimal protein expression, and inconsistent tissue regeneration.
- Current evidence and regulatory concerns do not yet support routine clinical use, and further high-quality human trials are required to establish efficacy, durability, and long-term safety.

## Hyaluronic acid

Endogenous hyaluronic acid (HA), also known as hyaluronan, is a glycosaminoglycan synthesized by three membrane enzymes: HA synthetases 1, 2, and 3 (HAS1, HAS2, and HAS3). It is formed through the repeated addition of disaccharide units of glucuronic acid and N-acetyl-D-glucosamine (106, 107). In normal human synovial fluid, HA is present as polymers with a molecular weight of approximately 4,000 kilodaltons. These polymers can retain up to 1,000 times their own weight in water, a property that underlies HA’s marked hydrophilicity and its important roles in joint lubrication and shock absorption (107).

### Sources and formulations

Exogenous HA may be produced by microbial enzymatic synthesis or extracted from rooster combs. Microbial production enables large-scale manufacturing, but bacterial strains may mutate or produce endotoxins that can trigger immunological hypersensitivity reactions. HA derived from rooster combs is contraindicated in patients with egg or poultry allergies and also poses challenges to achieving ultrapurification of the final product (107).

Commercial HA preparations also differ in several practical characteristics, including: concentration, typically ranging from 0.8 to 2.2%; ampoule volume; whether the product is cross-linked; molecular weight; and dosing regimen, which may range from a single injection to five weekly injections (107)

### Mechanisms of action

HA has mechanical, chondroprotective, and anti-inflammatory effects.

- The mechanical effects: HA acts as a lubricant, increases synovial fluid viscosity, absorbs shock, and stimulates endogenous HA production. These properties may help explain why clinical benefit can last for several months.
- The chondroprotective effects: HA may reduce enzymatic cartilage degradation and chondrocyte apoptosis while increasing proteoglycan/glycosaminoglycan and collagen synthesis, as well as chondrocyte proliferation.
- The anti-inflammatory effects: HA suppresses the expression of TNF-α, IL-1β, IL-6, IL-17, MMP, and PGE2 and reduces nitric oxide and free radicals.

These actions are mediated through binding to molecules such as hyaladherins, toll-like receptors, and layilin. Hyaladherins, or hyaluronan-binding proteins, include CD44, CD168/RHAMM, hyalectan, TSG-6, HABP1, and HABP2. They are involved in cell adhesion, extracellular matrix support, cell signaling, and cell migration, all of which contribute to tissue healing (32, 107–110).

Conversely, inflammatory mediators and reactive oxygen species reduce both HA production and molecular weight, thereby impairing its concentration, viscosity, and function (107).

### Molecular weight considerations

HA is commonly classified by molecular weight as follows: low molecular weight, < 800 kDa; medium molecular weight, 800–2,000 kDa; and high molecular weight, > 3,000 kDa (108–110).

Although higher-molecular-weight or cross-linked preparations tend to remain in the joint longer before enzymatic degradation, molecular weight has not consistently been shown to influence clinical outcomes (32, 107). Medium- to high-molecular-weight HA may more closely mimic endogenous HA; however, very high molecular weight preparations may reduce the availability of free binding sites on the cell surface and potentially impair endogenous HA biosynthesis (110).

High-molecular-weight HA also appears to exert anti-inflammatory effects by regulating immune-cell recruitment. In contrast, low-molecular-weight HA may promote angiogenesis and tissue remodeling but can also exert pro-inflammatory effects on chondrocytes (110).

### Clinical indications and overall evidence

Common musculoskeletal indications for HA include osteoarthritis (OA) and tendinopathies. Randomized controlled trials, systematic literature reviews, and meta-analyses have shown improvements in pain and function for knee OA, first carpometacarpal and shoulder OA. By contrast, evidence for hip, ankle, and elbow OA remains much less certain, although treatment is generally well tolerated and safe (32, 107, 111–120).

### Knee osteoarthritis: HA versus corticosteroids

A systematic literature review and meta-analysis comparing intra-articular HA with intra-articular corticosteroids (CS) in knee OA included 12 randomized controlled trials and 1,794 patients. Corticosteroids provided greater short-term pain relief, up to 1 month; however, by 6 months, the effect favored HA. Both treatments improved knee function to a similar extent and had comparable safety profiles, although HA was associated with more local adverse events (121).

### Knee osteoarthritis: HA plus PRP versus PRP alone

A systematic literature review and meta-analysis assessed the combination of intra-articular HA with platelet-rich plasma (PRP) versus PRP alone for knee OA. The analysis included 7 randomized controlled trials and 3 cohort studies, comprising 983 patients. Although the authors noted the lack of large, high-quality studies, the best available evidence suggested that combined treatment was superior to PRP alone for pain relief and functional improvement for up to 12 months (122).

### Glenohumeral osteoarthritis

Another systematic literature review and meta-analysis, including 15 randomized controlled trials and 1,023 patients, evaluated intra-articular HA in glenohumeral OA. HA was more effective than corticosteroid injections, and the combination of HA with physiotherapy provided greater pain relief than physiotherapy alone (108). Only one study compared HA with PRP in glenohumeral OA, and no difference was found between the two groups (108).

### Soft tissue indications

For soft tissue indications, HA is administered as soft tissue-adapted biocompatible hyaluronic acid (STABHA) (123). It has been used in ankle sprains, adhesive capsulitis, epicondylosis, and rotator cuff, Achilles, and patellar tendinopathies, with promising results. The strongest evidence currently relates to rotator cuff tendinopathy, including partial tendon tears and shoulder impingement syndrome (106, 123, 124).

A systematic literature review evaluating HA for soft tissue conditions compared it with placebo and other treatments, including PRP, CS, prolotherapy, and physical therapy. Across 19 randomized controlled trials involving 1,629 patients, HA was favored for pain relief in the short term (≤ 6 weeks) and mid-term (≤ 12 weeks), and for functional improvement up to 6 weeks. No major adverse effects were reported apart from injection-site discomfort (124).

Some condition-specific findings differed. For trigger finger, corticosteroids were superior to HA for short-term pain relief. In rotator cuff tendinosis, HA and extracorporeal shock wave therapy performed similarly, whereas HA was superior in Achilles tendinopathy. In patellar tendinopathy, HA performed similarly to PRP (124–127).

### Combination therapies and current recommendations

HA may be used alone or combined with other agents, including corticosteroids, PRP, ozone (O3), lactose-modified chitosan, and chondroitin sulfate. Some of these combinations may enhance HA efficacy in knee OA, although the supporting evidence remains limited (110, 128, 129).

Viscosupplementation Consensus Groups recommend intra-articular knee HA in selected situations, including:

- patients who previously responded well to HA injections;
- younger patients at high risk of OA progression;
- competitive athletes seeking to slow OA progression; and
- symptomatic adults with clinically and radiographically confirmed mild-to-moderate knee OA who have not yet received other therapies, have failed pharmacological or non-pharmacological treatment, or have had an incomplete response to previous therapies (130, 131).

### Safety

Reported adverse events include synovitis in patients with concomitant calcium pyrophosphate deposition disease and granulomatous synovitis.

### Remaining uncertainties

Despite the growing body of evidence, important questions remain regarding the optimal HA regimen, product origin, and ideal molecular weight (high versus low), largely because the available evidence is still insufficient to resolve these issues conclusively (32, 108, 109, 113, 132).

## Hydrodissection/brisement

Hydrodissection (HD), also known as percutaneous hydrostatic decompression or brisement, involves injecting a large volume (up to 15 mL) of 5% dextrose in water (D5W) around an affected nerve to separate it from nearby tissues. Other injectables besides D5W or normal saline (NS) have a volume limitation (CS, PRP, hyaluronidase), making them unsuitable for large-volume applications (133). HD may be associated with a local anesthetic (LA), with or without a CS, and may also be combined with PRP or HA (2).

**FIGURE 6:**
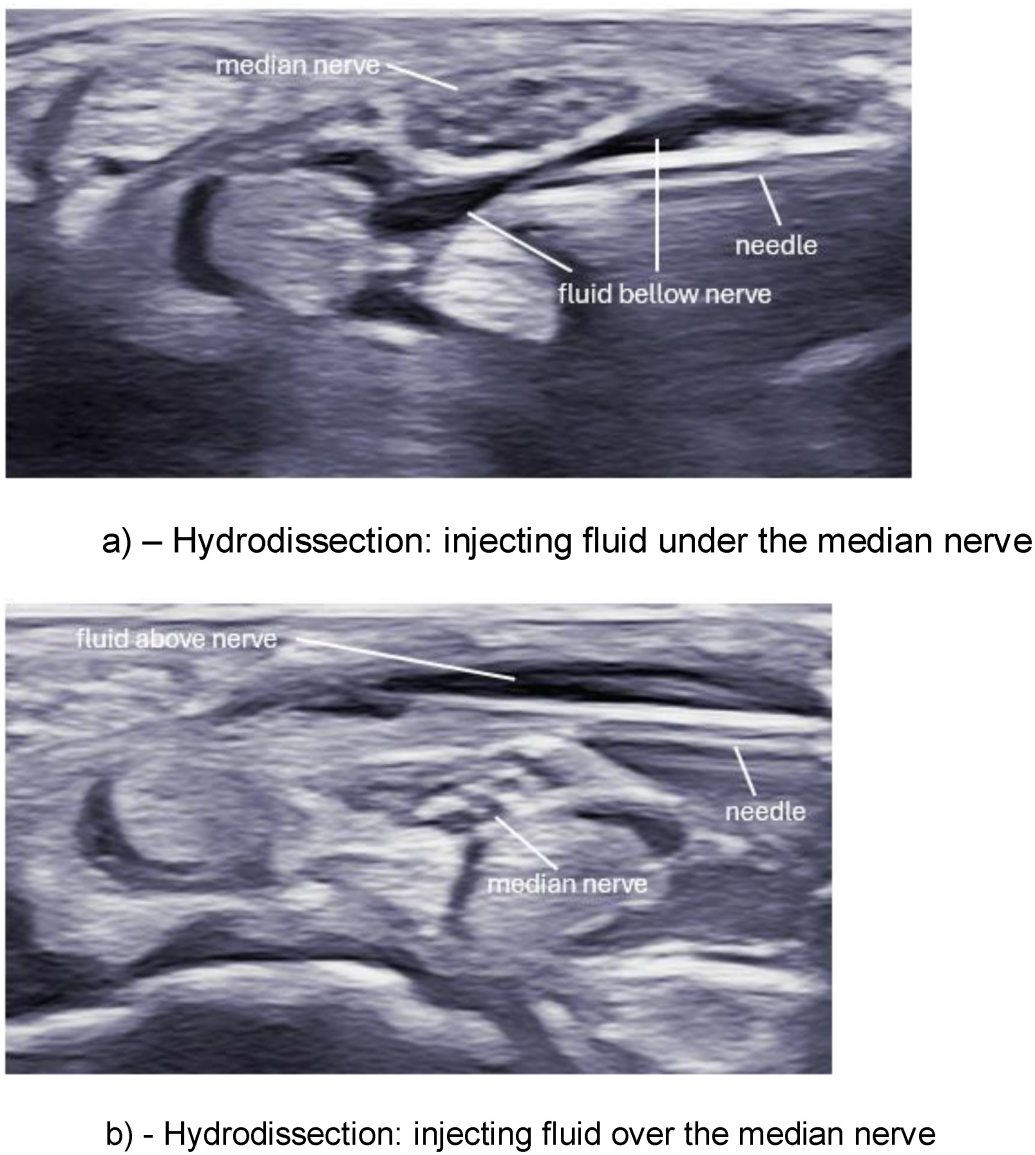
**(a)** Hydrodissection: injecting fluid under the median nerve. **(b)** Hydrodissection: injecting fluid over the median nerve.

A local anesthetic is used along the needle path, but care is taken not to inject it too close to the nerve to prevent temporary paralysis or nerve toxicity. Then D5W is injected to separate the nerve from the surrounding tissue. An in-plane technique having the nerve in a short-axis view is the best method for conducting an HD. The needle should first approach the deep surface of the nerve, having its bevel facing up. When fluid is injected, the resistance from the soft tissue guides the needle deeper, which helps prevent nerve injury. The procedure is repeated, this time with the needle nearing the nerve’s surface and its bevel facing downward (Figure 6). The procedure is complete when the nerve is seen surrounded by fluid. When a long section of the nerve is compressed, the same needle entry point is maintained, but both the probe and needle can be angled from the proximal to the distal end of the entrapment, repeating the hydrodissection at each site. Simultaneous HD proximal and distal to the entrapped nerve may be more effective than just one procedure, but it surely is more challenging technically. For carpal tunnel syndrome, which is the most common entrapment neuropathy, there is the recommendation of introducing the needle through the “transverse safe zone”, which is the space between the ulnar border of the median nerve and the radial border of the ulnar artery or through the “longitudinal safe zone”, which is the space between the distal part of the carpal annular ligament and the superficial palmar arch (133–136). Certain authors recommend combining the transverse ulnar approach with a longitudinal approach running from proximal to distal between the first and second carpal rows, employing a high volume (10 ml) of D5W (136).

The outcome of any HD will reflect the retention time and volume of the injectate, the entrapment grade and location, and the number of treatments, which may vary between 1 and 5, 1–4 weeks apart, in case of multiple treatments (133–135).

HD with D5W likely offers combined mechanical, pharmacological, and neuroregenerative benefits. The mechanical benefit may relate to the separation of the injured nerve from the surrounding soft tissues, lowering adhesions, allowing nerve mobilization, and releasing compressed nervi nervorum and vasa nervorum. The pharmacological effect of D5W, opposed to what happens with NS, results from a direct analgesic action while downregulating capsaicin-sensitive receptors, which are present in peripheral nerves, tendons, ligaments, and joints, impeding the release of SP and CGRP. Moreover, D5W may have an antinociceptive action through a decrease in C-fiber activation by reversing the local hypoglycemic status. Possible neuroregenerative effects of D5W explain the long-term benefits of the procedure (133, 134, 137). The pharmacological and neuroregenerative actions of D5W justify why D5W is more effective than NS in relieving symptoms of nerve entrapment and is the most common option in perineural HD (133, 135).

Common indications include entrapment neuropathies and neuromas, and less often Achilles tendinopathy and adhesive capsulitis (13, 133–136, 138, 139).

Concerning carpal tunnel syndrome, 4–10 mL D5W HD proved to be more effective than CS injections or 1–2 mL D5W HD, and a 10 ml volume of D5W seems to be the best option (136, 140–144). In ulnar nerve entrapment at the elbow, 5 ml D5W HD was as effective as an injection of CS plus NS, considering that the non-superiority of HD could be due to the particular anatomy of the ulnar tunnel, allowing the spilling of the injectate into other layers (145). HD with D5W on cervical root compression, thoracic outlet syndrome, or cervicogenic headache provided more than 50% relief, although sometimes only after several treatment courses (146). Several case reports were also published showing improvements after D5W HD in sensitive radial nerve entrapment (2 mL D5W), supinator syndrome, and superficial peroneal nerve entrapment (5 mL D5W each), meralgia paresthetica and pronator teres syndrome (10 ml D5W each), and radial nerve palsy (15 mL D5W), using 1–7 treatments (134). Other studies compared intra-articular CS injection with HD in shoulder adhesive capsulitis and found the two approaches equally effective (138). Additional HD targets include the stellate ganglion, brachial plexus, cervical nerve roots, and paravertebral spaces (135).

Specific AE may be due to anesthetic toxicity or allergy, if a LA is used in the context of the HD, or may be the result of nerve damage by the needle.

## Mesenchymal stromal cells

Stem cells can be classified by lineage commitment and differentiation potential into pluripotent stem cells (PPSCs) and multipotent stem cells (MPSCs). PPSCs, which include embryonic stem cells and induced pluripotent stem cells, can differentiate into any specialized cell type. In contrast, MPSCs, such as mesenchymal stem cells or mesenchymal stromal cells (MSCs), are limited to differentiation within a single germ layer (33). MSCs can differentiate into mesodermal tissues, including cartilage, bone, tendon, meniscus, muscle, and adipose tissue, which underpins their relevance in regenerative medicine. They also exert important paracrine effects and can modulate the local microenvironment. However, their multipotency has been demonstrated mainly *in vitro* rather than *in vivo*, although they can stimulate endogenous stem cell activity and secrete immunomodulatory bioactive factors that support tissue repair (147). MSCs are also thought to have a perivascular origin. This evidence underlies the shift in terminology from mesenchymal stem cells to mesenchymal stromal cells, although the term “medicinal signaling cells” has also been proposed while retaining the acronym MSCs (148).

MSCs may be derived from several sources:

• **Bone marrow-derived (BM-MSCs)**
  bone marrow aspirate (BMA)
  bone marrow aspirate concentrate (BMAC)
  mix (BMA+BMAC)
  hybrid bone marrow aspirate concentrate (HBMAC)
• **Adipose tissue-derived MSCs (AD-MSCs)**
  stromal vascular fraction (SVF)
  macroscopic fat tissue (Macro-FAT)
  microfragmented fat tissue (MFAT)
  nanofat tissue NFAT
• **Other sources of MSCs**

HBMAC combines BMAC with other orthobiologic products, such as PRP, platelet-rich fibrin (PRF), HA, growth factors (GFs), or gene therapy. Other sources of MSCs include amniotic-based products, umbilical cord, peripheral blood, skin, synovium, and the infrapatellar fat pad (33, 147, 149–153).

The International Society for Cellular Therapy proposed minimum criteria for cells to be classified as human MSCs: the cells must be able to differentiate *in vitro* into chondrogenic, osteogenic, or adipogenic tissue; they must express CD73, CD90, and CD105 markers, but not HLA-DR, CD11b, CD14, CD19, CD34, CD45, or CD79a; and they must be able to adhere to plastic under standard culture conditions (153, 154).

BM-MSCs are generally obtained from the patient’s bone marrow, most commonly harvested from the posterior superior iliac crest. Aspirated volumes should exceed 8 ml to improve consistency in cell yield. Bone marrow contains osteoblasts, osteoclasts, macrophages, endothelial progenitor cells, hematopoietic stem cells, and MSCs. In the case of BMAC, the aspirate is centrifuged to concentrate MSCs. The therapeutic effects of bone marrow-derived orthobiologic products are thought to result from both MSCs and hematopoietic stem cells. In addition, MSCs are believed to correspond to pericytes or perivascular cells, whose properties may vary according to the vascular niche in which they reside (148, 153).

AD-MSCs are obtained by means of a vacuum-locked syringe system or power-assisted liposuction, with or without prior administration of Klein’s or tumescent solution (lidocaine, normal saline, and epinephrine), to facilitate aspiration of abdominal subcutaneous fat. The harvested material is subsequently processed using techniques such as enzyme treatment, centrifugation, and washing (153). AD-MSCs may be prepared through three principal approaches: (1) SVF, obtained using enzymes such as collagenase, dispase, or trypsin, and composed of MSCs, endothelial progenitor cells, macrophages, pericytes, preadipocytes, and smooth muscle cells; (2) MFAT, produced through mechanical fragmentation of adipose tissue using metal beads or progressively smaller sieves combined with repeated saline washing, thereby avoiding enzymatic digestion; and 3) NFAT, generated by emulsification and filtration of the lipoaspirate, yielding a product that contains fragments of arterioles, venules, and capillaries, together with GFs, peptides, and cytokines (153).

In addition to AD-MSCs and BM-MSCs, several other MSC sources have been described, although these are limited by the relatively small amount of material that can be obtained. Amniotic-based products are derived from placental tissue obtained from donors undergoing elective cesarean section, after blunt dissection to separate the amniotic membrane from the chorion. The resulting product is stored in sterile vials following cryopreservation, freeze-drying, or gamma irradiation (153). Other perinatal products occasionally used include umbilical cord blood and Wharton’s jelly, both of which express several MSC-related genes and can be cultured and differentiated into multiple cell types (153).

MSCs are capable of self-renewal *in vivo* and display trophic, mitogenic, anti-fibrotic, anti-apoptotic, and immunomodulatory properties. They also retain the potential to differentiate into osteoblasts, myocytes, adipocytes, and chondrocytes (155). However, there is no definitive *in vivo* evidence that MSCs directly differentiate in sufficient numbers to replace damaged tissues, and studies have shown that fewer than 1% of injected cells remain after as little as 7 days (156, 157). Nonetheless, MSCs may be recruited by chemokines released from injured tissues. Once localized to the target site, they appear to coordinate reparative responses through autocrine and paracrine mechanisms, delivering GFs, cytokines, and other bioactive mediators such as angiopoietin 1 and 2, bone morphogenetic proteins, IL-6, VEGF, TGF-β, stromal-derived factor, stem-cell factor, and brain-derived neurotrophic factor. Through these mechanisms, MSCs may reduce fibrosis, apoptosis, and inflammation, including inhibition of pro-inflammatory cytokines such as IL-1, IL-6, and INF-γ, as well as modulation of type 1 macrophages, NK cells, and B and T leucocytes, while promoting tissue repair (147, 153).

Macro-FAT, MFAT, BMA (0.01–0.1%), BMAC (1–5%), Nano-FAT (2–5%), and SVF (15–30%) contain progressively increasing concentrations of MSCs, with Macro-FAT and MFAT used almost exclusively to provide structural support and cushioning (153, 158, 159).

The methods used to prepare BMAC are highly variable, which limits the comparability of treatments, and studies have so far failed to demonstrate any clear regenerative effect (27). Regarding autologous BM-MSCs, these usually need to be culture-expanded because of their limited number in a one-stage harvest protocol. Allogeneic MSCs are an alternative because they lack significant immunogenicity, which facilitates transplantation (147). In fact, allogeneic BM-MSCs are easy to obtain and allow the manufacture of larger amounts of product, although they also need to be culture-expanded (23, 160).

Adipose tissue has several advantages over other tissues as a source of MSCs, namely its abundance, ease of harvesting with minimal invasiveness, and a stable phenotype after many culture passages (23). Regarding SVF, it can be delivered in two different ways: through intra-articular injection of cells suspended in PRP or through surgical implantation. In the first case, because SVF is always suspended in a volume of PRP, there is no information regarding the regenerative effects of pure SVF in OA (23).

There are three ways of combining MSCs and PRP: using PRP lysates of bovine origin during laboratory expansion of MSCs; priming MSCs with PRP after cell expansion but before transplantation to improve their differentiation capabilities; and using PRP as a vehicle to confine MSCs to a chosen site and enhance the biological action of the implanted cells (151).

When administered intra-articularly to osteoarthritic joints, MSCs appear to adhere to damaged tissue surfaces and may protect against further cartilage degeneration through restoration of chondrocyte homeostasis and enhancement of matrix synthesis, potentially resulting in anatomical improvement, pain reduction, functional benefit, and a favorable safety profile. Similarly, MSCs have been proposed to enhance tendon healing by differentiating into tenocyte-like cells and secreting relevant growth factors and cytokines. Although direct differentiation into the desired target cell type, after injection into injured tissue, remains possible, the preponderance of evidence suggests that their principal mode of action is paracrine, promoting and coordinating regenerative and reparative processes. Accordingly, the primary therapeutic effects of MSCs are more likely to depend on their trophic and immunomodulatory properties than on direct replacement of damaged or absent tissue (31, 33, 34, 152, 153, 160).

Current indications for MSC-based therapies include osteoarthritis (OA), tendinopathies, focal cartilage defects, and spinal conditions. Among these, knee osteoarthritis has been the most extensively studied indication, with encouraging results reported in relation to pain, function, quality of life, and cartilage-related outcomes following intra-articular administration of BMAC, AD-MSCs, or peripheral blood-derived MSCs. In comparative studies, MSC-based therapies have frequently shown better outcomes than NS, CS, or HA, and adverse events have generally been mild and self-limited, most commonly transient pain and swelling (23, 27, 30, 31, 34, 149, 150, 155, 160). Combination therapy using MSCs with HA or PRP may yield better results than MSCs alone, although this finding has not been consistent across all studies (23, 27, 151, 152).

*In vitro* metabolic activity and viability of AD-MSCs appear to be adversely affected by the addition of local anesthetics such as lidocaine, bupivacaine, or ropivacaine at clinically relevant concentrations, with ropivacaine showing comparatively less toxicity. This detrimental effect appears to be proportional both to anesthetic concentration and to exposure time (161). Whether these findings translate into clinically relevant *in vivo* effects remains uncertain.

Despite promising findings regarding MSCs, several issues remain unresolved, including identification of the optimal MSC source, standardization of harvesting, culture expansion, and administration protocols (including dosage, volume, timing, and number of injections), and determination of the most appropriate stage of disease for treatment (150, 155, 156, 160). Some studies have reported contradictory results regarding high- versus low-dose AD-MSCs, as well as injection versus implantation strategies using BM-MSCs (37). A moderate cell dose (40 × 10^6^) appears to produce the most favorable outcomes in Kellgren grade ≥ 2 knee osteoarthritis. Noticeably, allogeneic MSCs may still be recognized by the host immune system, suggesting that lower doses could be preferable in some settings (23, 150). Nevertheless, there is strong evidence for the safety of MSC administration when performed by trained physicians, with the appropriate precautions, under image guidance, and using a sterile technique (148).

Overall, the inherent complexity of MSC-based therapies, together with the lack of high-level evidence and persistent methodological heterogeneity, continues to limit their routine incorporation as MIMSPs. Efforts to address some of these limitations are reflected in the ACH classification proposal, which seeks to standardize best practices for BM-MSC-based products (147).

## References

[1] Koski JM Hammer HB. Ultrasound-guided procedures: techniques and usefulness in controlling inflammation and disease progression. *Rheumatology.* (2012) 51:vii31–5. 10.1093/rheumatology/kes331 23230092

[2] Davidson J Jayaraman S. Guided interventions in musculoskeletal ultrasound: what’s the evidence? *Clin Radiol.* (2011) 66:140–52. 10.1016/j.crad.2010.09.006 21216330

[3] Robotti G Canepa MG Bortolotto C Draghi F. Interventional musculoskeletal US: an update on materials and methods. *J Ultrasound.* (2013) 16:45–55. 10.1007/s40477-013-0018-9 24294343 PMC3774911

[4] Saraiva F. Ultrasound-Guided synovial biopsy: a review. *Front Med.* (2021) 8:632224. 10.3389/fmed.2021.632224 33968950 PMC8100029

[5] Costa F Campanilho-Marques R Dourado E Bandeira M Roque R Fonseca JE et al. Efficacy and safety of ultrasound-guided needle muscle biopsy in the diagnosis of idiopathic inflammatory myopathies. *Rheumatology.* (2025) 64:5123–31. 10.1093/rheumatology/keaf241 40358534

[6] Epis O Bruschi E. Interventional ultrasound: a critical overview on ultrasound-guided injections and biopsies. *Clin Exp Rheumatol.* (2014) 32:S78–84. 24529311

[7] Epis O Iagnocco A Meenagh G Riente L Sedie AD Filippucci E et al. Ultrasound imaging for the rheumatologist. XLI. ultrasound-guided procedures. *Clin Exp Rheumatol.* (2008) 26:515–8. 18799078

[8] Sibbitt WL Peisajovich A Michael AA Park KS Sibbitt RR Band PA et al. Does sonographic needle guidance affect the clinical outcome of intraarticular injections? *J Rheumatol.* (2009) 36:1892–902. 10.3899/jrheum.090013 19648304

[9] Cipolletta E Filippucci E Incorvaia A Schettino M Smerilli G Di Battista J et al. Ultrasound-Guided procedures in rheumatology daily practice: feasibility, accuracy, and safety issues. *J Clin Rheumatol.* (2020) 27:226–31. 10.1097/RHU.0000000000001298 32000229

[10] Daniels EW Cole D Jacobs B Phillips SF. Existing evidence on ultrasound-guided injections in sports medicine. *Orthop J Sports Med.* (2018) 6:2325967118756576. 10.1177/2325967118756576 29511701 PMC5826008

[11] Dejaco C Machado PM Carubbi F Bosch P Terslev L Tamborrini G et al. EULAR points to consider for the use of imaging to guide interventional procedures in patients with rheumatic and musculoskeletal diseases (RMDs). *Ann Rheum Dis.* (2022) 81:760–7. 10.1136/annrheumdis-2021-221261 34893469

[12] Oo WM Linklater J Siddiq MAB Fu K Hunter DJ. Comparison of ultrasound guidance with landmark guidance for symptomatic benefits in knee, hip and hand osteoarthritis: systematic review and meta-analysis of randomised controlled trials. *Australas J Ultrasound Med.* (2024) 27:97–105. 10.1002/ajum.12386 38784696 PMC11109994

[13] Sibbitt WL Band PA Chavez-Chiang NR Delea SL Norton HE Bankhurst AD. A randomized controlled trial of the cost-effectiveness of ultrasound-guided intraarticular injection of inflammatory arthritis. *J Rheumatol.* (2011) 38:252–63. 10.3899/jrheum.100866 21078710

[14] Naredo E Rodriguez-Garcia SC Terslev L Martinoli C Klauser A Hartung W et al. The efsumb guidelines and recommendations for musculoskeletal ultrasound - Part II: joint pathologies, pediatric applications, and guided procedures. *Ultraschall Med.* (2022) 43:252–73. 10.1055/a-1640-9183 34734404

[15] Zadro J Rischin A Johnston RV Buchbinder R. Image-guided glucocorticoid injection versus injection without image guidance for shoulder pain. *Cochrane Database Syst Rev.* (2021) 8:CD009147. 10.1002/14651858.CD009147.pub3 34435661 PMC8407470

[16] Uson J Rodriguez-García SC Castellanos-Moreira R O’Neill TW Doherty M Boesen M et al. EULAR recommendations for intra-articular therapies. *Ann Rheum Dis.* (2021) 80:1299–305. 10.1136/annrheumdis-2021-220266 34035002 PMC8458067

[17] Draghi F Robotti G Jacob D Bianchi S. Interventional musculoskeletal ultrasonography: precautions and contraindications. *J Ultrasound.* (2010) 13:126–33. 10.1016/j.jus.2010.09.004 23396633 PMC3552654

[18] Kumar Sahu A Rath P Aggarwal B. Ultrasound-guided injections in musculo-skeletal system - an overview. *J Clin Orthop Trauma.* (2019) 10:669–73. 10.1016/j.jcot.2019.05.013 31316237 PMC6611943

[19] Rodriguez-García SC Castellanos-Moreira R Uson J Naredo E O’Neill TW Doherty M et al. Efficacy and safety of intra-articular therapies in rheumatic and musculoskeletal diseases: an overview of systematic reviews. *RMD Open.* (2021) 7:e001658. 10.1136/rmdopen-2021-001658 34103406 PMC8186751

[20] Tortora S Messina C Albano D Serpi F Corazza A Carrafiello G et al. Ultrasound-guided musculoskeletal interventional procedures around the elbow, hand and wrist excluding carpal tunnel procedures. *J Ultrason.* (2021) 21:e169–76. 10.15557/JoU.2021.0027 34258043 PMC8264808

[21] Albano D Messina C Gitto S Serpi F Sconfienza LM. Imaging-Guided musculoskeletal interventions in the lower limb. *Radiol Clin North Am.* (2023) 61:393–404. 10.1016/j.rcl.2022.10.012 36739153

[22] Tseng WC Chen YC Lee TM Chen WS. Plantar fasciitis: an updated review. *J Med Ultrasound.* (2023) 31:268–74. 10.4103/jmu.jmu_2_23 38264606 PMC10802877

[23] Mehranfar S Abdi Rad I Mostafav E Akbarzadeh A. The use of stromal vascular fraction (SVF), platelet-rich plasma (PRP) and stem cells in the treatment of osteoarthritis: an overview of clinical trials. *Artif Cells Nanomed Biotechnol.* (2019) 47:882–90. 10.1080/21691401.2019.1576710 30887856

[24] Rabago D Best TM Zgierska AE Zeisig E Ryan M Crane DA. systematic review of four injection therapies for lateral epicondylosis: prolotherapy, polidocanol, whole blood and platelet-rich plasma. *Br J Sports Med.* (2009) 43:471–81. 10.1136/bjsm.2008.052761 19028733 PMC2755040

[25] Giovannetti de Sanctis E Franceschetti E De Dona F Palumbo A Paciotti M Franceschi F. The efficacy of injections for partial rotator cuff tears: a systematic review. *J Clin Med.* (2021) 10:51. 10.3390/jcm10010051 33375716 PMC7795404

[26] Pavone V Vescio A Mobilia G Dimartino S Di Stefano G Culmone A et al. Conservative treatment of chronic achilles tendinopathy: a systematic review. *J Funct Morphol Kinesiol.* (2019) 4:46. 10.3390/jfmk4030046 33467361 PMC7739415

[27] Jones IA Togashi R Wilson ML Heckmann N Vangsness CT. Intra-articular treatment options for knee osteoarthritis. *Nat Rev Rheumatol.* (2019) 15:77–90. 10.1038/s41584-018-0123-4 30498258 PMC6390843

[28] Allen G Obradov M Chianca V Messina C Sconfienza LM. Ultrasound-guided musculoskeletal interventions for the most common hip and pelvis conditions: a step-by-step approach. *Semin Musculoskelet Radiol.* (2019) 23:e58–67. 10.1055/s-0039-1683965 31163510

[29] Lam KHS Wu YT Reeves KD Galluccio F Allam AE Peng PWH. Ultrasound-Guided interventions for carpal tunnel syndrome: a systematic review and meta-analyses. *Diagnostics.* (2023) 13:1138. 10.3390/diagnostics13061138 36980446 PMC10046938

[30] Beckmann NM Villamaria EE. Interventional therapies for osteoarthritis: an update. *AJR Am J Roentgenol.* (2022) 219:929–39. 10.2214/AJR.22.27548 35731097

[31] Migliore A Paoletta M Moretti A Liguori S Iolascon G. The perspectives of intra-articular therapy in the management of osteoarthritis. *Expert Opin Drug Deliv.* (2020) 17:1213–26. 10.1080/17425247.2020.1783234 32543240

[32] Huebner K Frank RM Getgood A. Ortho-Biologics for osteoarthritis. *Clin Sports Med.* (2019) 38:123–41. 10.1016/j.csm.2018.09.002 30466718

[33] Yang SM Chen WS. Conservative treatment of tendon injuries. *Am J Phys Med Rehabil.* (2019) 99:550–7. 10.1097/PHM.0000000000001345 31714291

[34] West WH Beutler AI Gordon CR. Regenerative injectable therapies: current evidence. *Curr Sports Med Rep.* (2020) 19:353–9. 10.1249/JSR.0000000000000751 32925374

[35] Malik KN Camp N Chan J Ballard M. Interventional techniques for the management of knee osteoarthritis: a literature review. *Cureus.* (2023) 15:e47133. 10.7759/cureus.47133 38022320 PMC10650933

[36] Nuhmani S. Injection therapies for patellar tendinopathy. *Phys Sportsmed.* (2019) 48:125–30. 10.1080/00913847.2019.1671143 31539489

[37] Shi WJ Tjoumakaris FP Lendner M Freedman KB. Biologic injections for osteoarthritis and articular cartilage damage: can we modify disease? *Phys Sportsmed.* (2017) 45:203–23. 10.1080/00913847.2017.1357421 28719231

[38] Chianca V Orlandi D Messina C Albano D Corazza A Rapisarda S et al. Interventional therapeutic procedures to treat degenerative and inflammatory musculoskeletal conditions: state of the art. *Radiol Med.* (2019) 124:1112–20. 10.1007/s11547-019-01018-8 30828775

[39] Oo WM Hunter DJ. Efficacy, safety, and accuracy of intra-articular therapies for hand osteoarthritis: current evidence. *Drugs Aging.* (2023) 40:1–20. 10.1007/s40266-022-00994-3 36633823

[40] de la Torre-Aboki J Uson J Pitsillidou I Vardanyan V Nikiphorou E Rodriguez-Garcia SC et al. Intra-articular therapies: patient preferences and professional practices in European countries. *Rheumatol Int.* (2022) 42:869–78. 10.1007/s00296-021-05045-5 34761277

[41] Messina C Sconfienza LM. Ultrasound-Guided percutaneous irrigation of calcific tendinopathy. *Semin Musculoskelet Radiol.* (2016) 20:409–13. 10.1055/s-0036-1594285 28002862

[42] De Conti G Marchioro U Dorigo A Boscolo N Vio S Trevisan M et al. Percutaneous ultrasound-guided treatment of shoulder tendon calcifications: clinical and radiological follow-up at 6 months(). *J Ultrasound.* (2010) 13:188–98. 10.1016/j.jus.2010.10.012 23396318 PMC3552650

[43] Lanza E Banfi G Serafini G Lacelli F Orlandi D Bandirali M et al. Ultrasound-guided percutaneous irrigation in rotator cuff calcific tendinopathy: what is the evidence? a systematic review with proposals for future reporting. *Eur Radiol.* (2015) 25:2176–83. 10.1007/s00330-014-3567-1 25583182

[44] Arirachakaran A Boonard M Yamaphai S Prommahachai A Kesprayura S Kongtharvonskul J. Extracorporeal shock wave therapy, ultrasound-guided percutaneous lavage, corticosteroid injection and combined treatment for the treatment of rotator cuff calcific tendinopathy: a network meta-analysis of RCTs. *Eur J Orthop Surg Traumatol.* (2016) 27:381–90. 10.1007/s00590-016-1839-y 27554465

[45] de Witte PB Kolk A Overes F Nelissen RGHH Reijnierse M. Rotator cuff calcific tendinitis: ultrasound-guided needling and lavage versus subacromial corticosteroids: five-year outcomes of a randomized controlled trial. *Am J Sports Med.* (2017) 45:3305–14. 10.1177/0363546517721686 28898104

[46] Sconza C Palloni V Lorusso D Guido F Farì G Tognolo L et al. Ultrasound-guided percutaneous lavage for the treatment of rotator cuff calcific tendinopathy: a systematic review with meta-analysis of randomized controlled trials. *Eur J Phys Rehabil Med.* (2024) 60:995–1008. 10.23736/S1973-9087.24.08544-7 39382530 PMC11729717

[47] Diercks R Bron C Dorrestijn O Meskers C Naber R de Ruiter T et al. Guideline for diagnosis and treatment of subacromial pain syndrome: a multidisciplinary review by the dutch orthopaedic association. *Acta Orthop.* (2014) 85:314–22. 10.3109/17453674.2014.920991 24847788 PMC4062801

[48] Godoy IR Donahue DM Torriani M. Botulinum toxin injections in musculoskeletal disorders. *Semin Musculoskelet Radiol.* (2016) 20:441–52. 10.1055/s-0036-1594284 28002866

[49] Moore C Hulsopple C Boyce B. Utilization of botulinum toxin for musculoskeletal disorders. *Curr Sports Med Rep.* (2020) 19:217–22. 10.1249/JSR.0000000000000720 32516192

[50] Poenaru D Sandulescu MI Cinteza D. Pain modulation in chronic musculoskeletal disorders: botulinum toxin, a descriptive analysis. *Biomedicines.* (2023) 11:1888. 10.3390/biomedicines11071888 37509527 PMC10376837

[51] Chang KV Chiu YH Wu WT Hsu PC Özçakar L. Botulinum toxin injections for shoulder and upper limb pain: a narrative review. *Pain Manag.* (2020) 10:411–20. 10.2217/pmt-2020-0015 33073703

[52] Battista S Buzzatti L Gandolfi M Finocchi C Falsiroli Maistrello L Viceconti A et al. The use of botulinum toxin a as an adjunctive therapy in the management of chronic musculoskeletal pain: a systematic review with meta-analysis. *Toxins.* (2021) 13:640. 10.3390/toxins13090640 34564644 PMC8473399

[53] Sconza C Leonardi G Carfì C Kon E Respizzi S Scaturro D et al. Intra-Articular injection of botulinum toxin for the treatment of knee osteoarthritis: a systematic review of randomized controlled trials. *Int J Mol Sci.* (2023) 24:1486. 10.3390/ijms24021486 36674999 PMC9863806

[54] Kumar R. Therapeutic use of botulinum toxin in pain treatment. *Neuronal Signal.* (2018) 2:NS20180058. 10.1042/NS20180058 32714587 PMC7373233

[55] Ammitzbøll-Danielsen M Østergaard M Fana V Glinatsi D Døhn UM Ørnbjerg LM et al. Intramuscular versus ultrasound-guided intratenosynovial glucocorticoid injection for tenosynovitis in patients with rheumatoid arthritis: a randomised, double-blind, controlled study. *Ann Rheum Dis.* (2016) 76:666–72. 10.1136/annrheumdis-2016-209840 27604532

[56] Guermazi A Hunter DJ Kloppenburg M. Debate: Intra-articular steroid injections for osteoarthritis - harmful or helpful?✮✮. *Osteoarthr Imaging.* (2023) 3:100163. 10.1016/j.ostima.2023.100163 38313846 PMC10836165

[57] Raynauld JP Buckland-Wright C Ward R Choquette D Haraoui B Martel-Pelletier J et al. Safety and efficacy of long-term intraarticular steroid injections in osteoarthritis of the knee: a randomized, double-blind, placebo-controlled trial. *Arthritis Rheum.* (2003) 48:370–7. 10.1002/art.10777 12571845

[58] Jüni P Hari R Rutjes AW Fischer R Silletta MG Reichenbach S et al. Intra-articular corticosteroid for knee osteoarthritis. *Cochrane Database Syst Rev.* (2015) 2015:CD005328. 10.1002/14651858.CD005328.pub3 26490760 PMC8884338

[59] Cormier G Denis A Leske C Varin S Dimet J Planche L et al. Corticosteroids with or without hyaluronic acid injection in the treatment of trapeziometacarpal osteoarthritis: the randomised multicentre RHIZART trial study protocol (abstract). *Arthritis Rheumatol.* (2022) 74:e022553. 10.1136/bmjopen-2018-022553 30782680 PMC6340006

[60] Zhong HM Zhao GF Lin T Zhang XX Li XY Lin JF et al. Intra-Articular steroid injection for patients with hip osteoarthritis: a systematic review and meta-analysis. *Biomed Res Int.* (2020) 2020:6320154. 10.1155/2020/6320154 32185212 PMC7060863

[61] Kullenberg B Runesson R Tuvhag R Olsson C Resch S. Intra-articular corticosteroid injections: pain relief in osteoarthritis of the hip? *J Rheumatol.* (2004) 31:2265–8. 15517641

[62] Najm A Alunno A Gwinnutt JM Weill C Berenbaum F. Efficacy of intra-articular corticosteroid injections in knee osteoarthritis: A systematic review and meta-analysis of randomized controlled trials. *Joint Bone Spine.* (2021) 88:105198. 10.1016/j.jbspin.2021.105198 33901659

[63] Wang S Wang X Liu Y Sun X Tang Y. Ultrasound-guided intra-articular triamcinolone acetonide injection for treating refractory small joints arthritis of rheumatoid arthritis patients. *Medicine.* (2019) 98:e16714. 10.1097/MD.0000000000016714 31415364 PMC6831351

[64] Eder L Chandran V Ueng J Bhella S Lee KA Rahman P et al. Predictors of response to intra-articular steroid injection in psoriatic arthritis. *Rheumatology.* (2010) 49:1367–73. 10.1093/rheumatology/keq102 20388640

[65] Hetland ML Østergaard M Ejbjerg B Jacobsen S Stengaard-Pedersen K Junker P et al. Short- and long-term efficacy of intra-articular injections with betamethasone as part of a treat-to-target strategy in early rheumatoid arthritis: impact of joint area, repeated injections, MRI findings, anti-CCP, IgM-RF and CRP. *Ann Rheum Dis.* (2012) 71:851–6. 10.1136/annrheumdis-2011-200632 22302316

[66] Freire V Bureau NJ. Injectable corticosteroids: take precautions and use caution. *Semin Musculoskelet Radiol.* (2016) 20:401–8. 10.1055/s-0036-1594286 28002861

[67] Phillips M Vannabouathong C Devji T Patel R Gomes Z Patel A et al. Differentiating factors of intra-articular injectables have a meaningful impact on knee osteoarthritis outcomes: a network meta-analysis. *Knee Surg Sports Traumatol Arthrosc.* (2020) 28:3031–9. 10.1007/s00167-019-05763-1 31897550 PMC7471203

[68] Ran J Yang X Ren Z Wang J Dong H. Comparison of intra-articular hyaluronic acid and methylprednisolone for pain management in knee osteoarthritis: a meta-analysis of randomized controlled trials. *Int J Surg.* (2018) 53:103–10. 10.1016/j.ijsu.2018.02.065 29574247

[69] Duarte-Monteiro AM Dourado E Fonseca JE Saraiva F. Safety of glucocorticoid injections. state of the art. *ARP Rheumatol.* (2023) 1:64–73. 37042846

[70] Berthelot JM Tortellier L Guillot P Prost A Caumon JP Glemarec J et al. Tachon’s syndrome (suracute back and/or thoracic pain following local injections of corticosteroids). A report of 318 French cases. *Joint Bone Spine.* (2005) 72:66–8. 10.1016/j.jbspin.2004.01.005 15681251

[71] Stephens MB Beutler AL O’Connor FG. Musculoskeletal injections: a review of the evidence. *AM Fam Physician.* (2008) 78:971–6. 18953975

[72] Habib GS Saliba W Nashashibi M. Local effects of intra-articular corticosteroids. *Clin Rheumatol.* (2010) 29:347–56. 10.1007/s10067-009-1357-y 20101428

[73] Hess SR O’Connell RS Bednarz CP Waligora AC Golladay GJ Jiranek WA. Association of rapidly destructive osteoarthritis of the hip with intra-articular steroid injections. *Arthroplast Today.* (2018) 4:205–9. 10.1016/j.artd.2017.12.002 29896554 PMC5994787

[74] Tiwari A Karkhur Y Keeney JA Aggarwal A. Rapid destructive osteoarthritis of the hip after intra-articular steroid injection. *Arthroplast Today.* (2018) 4:184–6. 10.1016/j.artd.2018.01.002 29896550 PMC5994788

[75] Simeone FJ Vicentini JRT Bredella MA Chang CY. Are patients more likely to have hip osteoarthritis progression and femoral head collapse after hip steroid/anesthetic injections? A retrospective observational study. *Skeletal Radiol.* (2019) 48:1417–26. 10.1007/s00256-019-03189-x 30840099

[76] Petersen S Hansen I Andreasen R. Low frequency of septic arthritis after arthrocentesis and intra-articular glucocorticoid injection. *Scand J Rheumatol.* (2019) 48:393–7. 10.1080/03009742.2019.1584329 31146626

[77] Jayaseelan DJ Faller BT Avery MH. The utilization and effects of filiform dry needling in the management of tendinopathy: a systematic review. . *Physiother Theory Pract.* (2021) 38:1876–88. 10.1080/09593985.2021.1920076 33904812

[78] Gómez-Chiguano GF Navarro-Santana MJ Cleland JA Arias-Buría JL Fernández-de-Las-Peñas C Ortega-Santiago R et al. Effectiveness of ultrasound-guided percutaneous electrolysis for musculoskeletal pain: a systematic review and meta-analysis. *Pain Med.* (2020) 22:1055–71. 10.1093/pm/pnaa342 33155055

[79] Navarro-Santana MJ Gómez-Chiguano GF Cleland JA Arias-Buría JL Fernández-de-Las-Peñas C Plaza-Manzano G. Effects of trigger point dry needling for nontraumatic shoulder pain of musculoskeletal origin: a systematic review and meta-analysis. *Phys Ther.* (2021) 101:zaa216. 10.1093/ptj/pzaa216 33340405

[80] Dach F Ferreira KS. Treating myofascial pain with dry needling: a systematic review for the best evidence-based practices in low back pain. *Arq Neuropsiquiatr.* (2023) 81:1169–78. 10.1055/s-0043-1777731 38157883 PMC10756779

[81] Krey D Borchers J McCamey K. Tendon needling for treatment of tendinopathy: a systematic review. *Phys Sportsmed.* (2015) 43:80–6. 10.1080/00913847.2015.1004296 25613418

[82] Settergren R. Treatment of supraspinatus tendinopathy with ultrasound guided dry needling. *J Chiropr Med.* (2013) 12:26–9. 10.1016/j.jcm.2012.11.002 23997721 PMC3610946

[83] Gattie E Cleland JA Snodgrass S. The effectiveness of trigger point dry needling for musculoskeletal conditions by physical therapists: a systematic review and meta-analysis. *J Orthop Sports Phys Ther.* (2017) 47:133–49. 10.2519/jospt.2017.7096 28158962

[84] Gattie E Cleland JA Pandya J Snodgrass S. Dry needling adds no benefit to the treatment of neck pain: a sham-controlled randomized clinical trial with 1-year follow-up. *J Orthop Sports Phys Ther.* (2021) 51:37–45. 10.2519/jospt.2021.9864 33383999

[85] Para-García G García-Muñoz AM López-Gil JF Ruiz-Cárdenas JD García-Guillén AI López-Román FJ et al. Dry Needling alone or in combination with exercise therapy versus other interventions for reducing pain and disability in subacromial pain syndrome: a systematic review and meta-analysis. *Int J Environ Res Public Health.* (2022) 19:10961. 10.3390/ijerph191710961 36078676 PMC9518516

[86] Navarro-Santana MJ Sanchez-Infante J Gómez-Chiguano GF Cleland JA López-de-Uralde-Villanueva I Fernández-de-Las-Peñas C et al. Effects of trigger point dry needling on lateral epicondylalgia of musculoskeletal origin: a systematic review and meta-analysis. *Clin Rehabil.* (2020) 34:1327–40. 10.1177/0269215520937468 32576044

[87] Llurda-Almuzara L Labata-Lezaun N Meca-Rivera T Navarro-Santana MJ Cleland JA Fernández-de-Las-Peñas C et al. Is dry needling effective for the management of plantar heel pain or plantar fasciitis? an updated systematic review and meta-analysis. *Pain Med.* (2021) 22:1630–41. 10.1093/pm/pnab114 33760098

[88] James SL Ali K Pocock C Robertson C Walter J Bell J et al. Ultrasound guided dry needling and autologous blood injection for patellar tendinosis. *Br J Sports Med.* (2007) 41: 518–21; discussion 522. 10.1136/bjsm.2006.034686 17387140 PMC2465422

[89] Finnoff JT Fowler SP Lai JK Santrach PJ Willis EA Sayeed YA et al. Treatment of chronic tendinopathy with ultrasound-guided needle tenotomy and platelet-rich plasma injection. *PM R.* (2011) 3:900–11. 10.1016/j.pmrj.2011.05.015 21872551

[90] Suresh SP Ali KE Jones H Connell DA. Medial epicondylitis: is ultrasound guided autologous blood injection an effective treatment? *Br J Sports Med.* (2006) 40:935–9; discussion 939. 10.1136/bjsm.2006.029983 16990441 PMC2465032

[91] Stoychev V Finestone AS Kalichman L. Dry needling as a treatment modality for tendinopathy: a narrative review. *Curr Rev Musculoskelet Med.* (2020) 13:133–40. 10.1007/s12178-020-09608-0 31942676 PMC7083985

[92] Valera-Calero JA Plaza-Manzano G Rabanal-Rodríguez G Díaz-Arribas MJ Kobylarz MD Buffet-García J et al. Current state of dry needling practices: a comprehensive analysis on use, training, and safety. *Medicina.* (2024) 60:1869. 10.3390/medicina60111869 39597054 PMC11596814

[93] Connell DA Ali KE Ahmad M Lambert S Corbett S Curtis M. Ultrasound-guided autologous blood injection for tennis elbow. *Skeletal Radiol.* (2006) 35:371–7. 10.1007/s00256-006-0081-9 16552606

[94] Evans CH Ghivizzani SC Robbins PD. Gene delivery to joints by intra-articular injection. *Hum Gene Ther.* (2018) 29:2–14. 10.1089/hum.2017.181 29160173 PMC5773261

[95] Deviatkin AA Vakulenko YA Akhmadishina LV Tarasov VV Beloukhova MI Zamyatnin AA et al. Emerging concepts and challenges in rheumatoid arthritis gene therapy. *Biomedicines.* (2020) 8:9. 10.3390/biomedicines8010009 31936504 PMC7168286

[96] Evans C. Editorial: arthritis gene therapy using interleukin-1 receptor antagonist. *Arthritis Rheumatol.* (2018) 70:1699–701. 10.1002/art.40675 30035385 PMC6203597

[97] Bellavia D Veronesi F Carina V Costa V Raimondi L De Luca A et al. Gene therapy for chondral and osteochondral regeneration: is the future now? *Cell Mol Life Sci.* (2018) 75:649–67. 10.1007/s00018-017-2637-3 28864934 PMC11105387

[98] Evans CH Ghivizzani SC Robbins PD. Arthritis gene therapy and its tortuous path into the clinic. *Transl Res.* (2013) 161:205–16. 10.1016/j.trsl.2013.01.002 23369825 PMC3602127

[99] Rodriguez-Merchan EC Valentino LA. The role of gene therapy in cartilage repair. *Arch Bone Jt Surg.* (2019) 7:79–90. 31211186 PMC6510927

[100] Ilaltdinov AW Gong Y Leong DJ Gruson KI Zheng D Fung DT et al. Advances in the development of gene therapy, noncoding RNA, and exosome-based treatments for tendinopathy. *Ann N Y Acad Sci.* (2020) 1490:3–12. 10.1111/nyas.14382 32501571

[101] Uzieliene I Kalvaityte U Bernotiene E Mobasheri A. Non-viral gene therapy for osteoarthritis. *Front Bioeng Biotechnol.* (2020) 8:618399. 10.3389/fbioe.2020.618399 33520968 PMC7838585

[102] Watkins LR Chavez RA Landry R Fry M Green-Fulgham SM Coulson JD et al. Targeted interleukin-10 plasmid DNA therapy in the treatment of osteoarthritis: toxicology and pain efficacy assessments. *Brain Behav Immun.* (2020) 90:155–66. 10.1016/j.bbi.2020.08.005 32800926

[103] Tsitrouli Z Akritidou MA Genitsaris S Willigen GV. Treatment of rheumatoid arthritis with gene therapy applications: biosafety and bioethical considerations. *BioTech.* (2021) 10:11. 10.3390/biotech10030011 35822765 PMC9245461

[104] Zhang Q Yu FX Wu YL Yang CY Liu NC Zhu X et al. Novel gene therapy for rheumatoid arthritis with single local injection: adeno-associated virus-mediated delivery of A20/TNFAIP3. *Mil Med Res.* (2022) 9:34. 10.1186/s40779-022-00393-0 35729676 PMC9210758

[105] Lim CL Lee YJ Cho JH Choi H Lee B Lee MC et al. Immunogenicity and immunomodulatory effects of the human chondrocytes, hChonJ. *BMC Musculoskelet Disord.* (2017) 18:199. 10.1186/s12891-017-1547-8 28521800 PMC5437658

[106] Oliva F Marsilio E Asparago G Frizziero A Berardi AC Maffulli N. The impact of hyaluronic acid on tendon physiology and its clinical application in tendinopathies. *Cells.* (2021) 10:3081. 10.3390/cells10113081 34831304 PMC8625461

[107] Bowden DJ Byrne CA Alkhayat A Eustace SJ Kavanagh EC. Injectable viscoelastic supplements: a review for radiologists. *AJR Am J Roentgenol.* (2017) 209:883–8. 10.2214/AJR.17.17847 28937277

[108] Familiari F Ammendolia A Rupp MC Russo R Pujia A Montalcini T et al. Efficacy of intra-articular injections of hyaluronic acid in patients with glenohumeral joint osteoarthritis: a systematic review and meta-analysis. *J Orthop Res.* (2023) 41:2345–58. 10.1002/jor.25648 37314198

[109] da Costa SR da Mota E Albuquerque RF Helito CP Camanho GL. The role of viscosupplementation in patellar chondropathy. *Ther Adv Musculoskelet Dis.* (2021) 13:1759720X211015005. 10.1177/1759720X211015005 34035839 PMC8127754

[110] Costa FR Costa Marques MR Costa VC Santos GS Martins RA Santos MDS et al. Intra-Articular hyaluronic acid in osteoarthritis and tendinopathies: molecular and clinical approaches. *Biomedicines.* (2023) 11:1061. 10.3390/biomedicines11041061 37189679 PMC10135595

[111] Miller LE Block JE. US-Approved intra-articular hyaluronic acid injections are safe and effective in patients with knee osteoarthritis: systematic review and meta-analysis of randomized, saline-controlled trials. *Clin Med Insights Arthritis Musculoskelet Disord.* (2013) 6:57–63. 10.4137/CMAMD.S12743 24027421 PMC3767581

[112] Bhandari M Bannuru RR Babins EM Martel-Pelletier J Khan M Raynauld JP et al. Intra-articular hyaluronic acid in the treatment of knee osteoarthritis: a Canadian evidence-based perspective. *Ther Adv Musculoskelet Dis.* (2017) 9:231–46. 10.1177/1759720X17729641 28932293 PMC5600311

[113] Bannuru RR Vaysbrot EE Sullivan MC McAlindon TE. Relative efficacy of hyaluronic acid in comparison with NSAIDs for knee osteoarthritis: a systematic review and meta-analysis. *Semin Arthritis Rheum.* (2014) 43:593–9. 10.1016/j.semarthrit.2013.10.002 24216297

[114] Bellamy N Campbell J Robinson V Gee T Bourne R Wells G. Viscosupplementation for the treatment of osteoarthritis of the knee. *Cochrane Database Syst Rev.* (2006) 2006:CD005321. 10.1002/14651858.CD005321.pub2 16625635 PMC8884110

[115] Campbell KA Erickson BJ Saltzman BM Mascarenhas R Bach BR Cole BJ et al. Is local viscosupplementation injection clinically superior to other therapies in the treatment of osteoarthritis of the knee: a systematic review of overlapping meta-analyses. *Arthroscopy.* (2015) 31: 2036–45.e14. 10.1016/j.arthro.2015.03.030 25998016

[116] Qvistgaard E Christensen R Torp-Pedersen S Bliddal H. Intra-articular treatment of hip osteoarthritis: a randomized trial of hyaluronic acid, corticosteroid, and isotonic saline. *Osteoarthritis Cartilage.* (2006) 14:163–70. 10.1016/j.joca.2005.09.007 16290043

[117] Richette P Ravaud P Conrozier T Euller-Ziegler L Mazières B Maugars Y et al. Effect of hyaluronic acid in symptomatic hip osteoarthritis: a multicenter, randomized, placebo-controlled trial. *Arthritis Rheum.* (2009) 60:824–30. 10.1002/art.24301 19248105

[118] Colen S Geervliet P Haverkamp D Van Den Bekerom MP. Intra-articular infiltration therapy for patients with glenohumeral osteoarthritis: a systematic review of the literature. *Int J Shoulder Surg.* (2014) 8:114–21. 10.4103/0973-6042.145252 25538430 PMC4262866

[119] Trellu S Dadoun S Berenbaum F Fautrel B Gossec L. Intra-articular injections in thumb osteoarthritis: a systematic review and meta-analysis of randomized controlled trials. *Joint Bone Spine.* (2015) 82:315–9. 10.1016/j.jbspin.2015.02.002 25776442

[120] Witteveen AG Hofstad CJ Kerkhoffs GM. Hyaluronic acid and other conservative treatment options for osteoarthritis of the ankle. *Cochrane Database Syst Rev.* (2015) 2015:CD010643. 10.1002/14651858.CD010643.pub2 26475434 PMC9254328

[121] He WW Kuang MJ Zhao J Sun L Lu B Wang Y et al. Efficacy and safety of intraarticular hyaluronic acid and corticosteroid for knee osteoarthritis: a meta-analysis. *Int J Surg.* (2017) 39:95–103. 10.1016/j.ijsu.2017.01.087 28137554

[122] Aw AAL Leeu JJ Tao X Bin Abd Razak HR. Comparing the efficacy of dual Platelet-Rich Plasma (PRP) and Hyaluronic Acid (HA) therapy with PRP-alone therapy in the treatment of knee osteoarthritis: a systematic review and meta-analysis. *J Exp Orthop.* (2021) 8:101. 10.1186/s40634-021-00415-1 34735663 PMC8569119

[123] Cipolletta E Mashadi Mirza R Di Matteo A Di Carlo M Grassi W Filippucci E. Clinical efficacy of ultrasound-guided hyaluronic acid injections in patients with supraspinatus tendon tear. *Clin Exp Rheumatol.* (2021) 39:769–74. 10.55563/clinexprheumatol/cyiyy3 32896264

[124] Khan M Shanmugaraj A Prada C Patel A Babins E Bhandari M. The role of hyaluronic acid for soft tissue indications: a systematic review and meta-analysis. *Sports Health.* (2023) 15:86–96. 10.1177/19417381211073316 35114853 PMC9808833

[125] Frizziero A Vittadini F Barazzuol M Gasparre G Finotti P Meneghini A et al. Extracorporeal shockwaves therapy versus hyaluronic acid injection for the treatment of painful non-calcific rotator cuff tendinopathies: preliminary results. *J Sports Med Phys Fitness.* (2017) 57:1162–8. 10.23736/S0022-4707.16.06408-2 27070534

[126] Lynen N De Vroey T Spiegel I Van Ongeval F Hendrickx NJ Stassijns G. Comparison of peritendinous hyaluronan injections versus extracorporeal shock wave therapy in the treatment of painful achilles’ tendinopathy: a randomized clinical efficacy and safety study. *Arch Phys Med Rehabil.* (2017) 98:64–71. 10.1016/j.apmr.2016.08.470 27639439

[127] Kaux J Bornheim S Dardenne E Deroisyr R Samsona J Roberjot M et al. Comparison between platelet-rich plasma injections and hyaluronic acid injections in the treatment of patellar tendinopathies: a randomized trial. *Muscles Ligaments Tendons J.* (2019) 9:322–7. 10.32098/mltj.03.2019.03

[128] Costa FR Santos MDS Martins RA Costa CB Hamdan PC Da Silva MB et al. The synergistic effects of hyaluronic acid and platelet-rich plasma for patellar chondropathy. *Biomedicines.* (2024) 12:6. 10.3390/biomedicines12010006 38275367 PMC10813186

[129] Pavelka K Horváth R Hurnáková J Saracino L Giordan N Procházková L et al. Clinical effectiveness and safety of intra-articular injection of HYALGO in the management of knee osteoarthritis symptoms: a multicenter prospective study. *J Clin Orthop Trauma.* (2021) 19:75–80. 10.1016/j.jcot.2021.05.009 34099970 PMC8165427

[130] Raman R Henrotin Y Chevalier X Migliore A Jerosch J Montfort J et al. Decision algorithms for the retreatment with viscosupplementation in patients suffering from knee osteoarthritis: recommendations from the EUROpean viscosupplementation consensus group (EUROVISCO). *Cartilage.* (2018) 9:263–75. 10.1177/1947603517693043 29110511 PMC6042033

[131] Bhadra AK Altman R Dasa V Myrick K Rosen J Vad V et al. Appropriate use criteria for hyaluronic acid in the treatment of knee osteoarthritis in the United States. *Cartilage.* (2017) 8:234–54. 10.1177/1947603516662503 28618868 PMC5625860

[132] Parisi S Ditto MC Priora M Borrelli R Laganà A Peroni CL et al. Ultrasound-guided intra-articular injection: efficacy of hyaluronic acid compared to glucocorticoid in the treatment of knee osteoarthritis. *Minerva Med.* (2019) 110:515–23. 10.23736/S0026-4806.19.06190-1 31965779

[133] Lam KHS Hung CY Chiang YP Onishi K Su DCJ Clark TB et al. Ultrasound-Guided nerve hydrodissection for pain management: rationale. methods, current literature, and theoretical mechanisms. *J Pain Res.* (2020) 13:1957–68. 10.2147/JPR.S247208 32801851 PMC7414936

[134] Wu YT Wu CH Lin JA Su DC Hung CY Lam SKH. Efficacy of 5% dextrose water injection for peripheral entrapment neuropathy: a narrative review. *Int J Mol Sci.* (2021) 22:12358. 10.3390/ijms222212358 34830240 PMC8621462

[135] Chang KV Wu WT Özçakar L. Ultrasound imaging and guidance in peripheral nerve entrapment: hydrodissection highlighted. *Pain Manag.* (2020) 10:97–106. 10.2217/pmt-2019-0056 32162601

[136] Kumar N Chandan SK Jalan D Sinha S Jaiswal B Singh DK. Ultrasound-guided interventions in primary carpal tunnel syndrome: perineural injection to thread carpal tunnel release. *Br J Radiol.* (2023) 96:20230552. 10.1259/bjr.20230552 37660684 PMC10546448

[137] Sveva V Farì G Fai A Savina A Viva MG Agostini F et al. Safety and efficacy of ultrasound-guided perineural hydrodissection as a minimally invasive treatment in carpal tunnel syndrome: a systematic review. *J Pers Med.* (2024) 14:154. 10.3390/jpm14020154 38392587 PMC10890373

[138] Redler LH Dennis ER. Treatment of adhesive capsulitis of the shoulder. *J Am Acad Orthop Surg.* (2019) 27:e544–54. 10.5435/JAAOS-D-17-00606 30632986

[139] Cormick W. Ultrasound, tendon pain and tendon disease. what’s new and what’s around the corner. *Sound Effects.* (2009) 2:12–5.

[140] Wu YT Ho TY Chou YC Ke MJ Li TY Tsai CK et al. Six-month efficacy of perineural dextrose for carpal tunnel syndrome: a prospective, randomized, double-blind, controlled trial. *Mayo Clin Proc.* (2017) 92:1179–89. 10.1016/j.mayocp.2017.05.025 28778254

[141] Wu YT Ke MJ Ho TY Li TY Shen YP Chen LC. Randomized double-blinded clinical trial of 5% dextrose versus triamcinolone injection for carpal tunnel syndrome patients. *Ann Neurol.* (2018) 84:601–10. 10.1002/ana.25332 30187524

[142] Li TY Chen SR Shen YP Chang CY Su YC Chen LC et al. Long-term outcome after perineural injection with 5% dextrose for carpal tunnel syndrome: a retrospective follow-up study. *Rheumatology.* (2021) 60:881–7. 10.1093/rheumatology/keaa361 32856082

[143] Lin MT Liao CL Hsiao MY Hsueh HW Chao CC Wu CH. Volume matters in ultrasound-guided perineural dextrose injection for carpal tunnel syndrome: a randomized. double-blinded, three-arm trial. *Front Pharmacol.* (2020) 11:625830. 10.3389/fphar.2020.625830 33391002 PMC7773892

[144] Lin MT Liu IC Syu WT Kuo PL Wu CH. Effect of perineural injection with different dextrose volumes on median nerve size, elasticity and mobility in hands with carpal tunnel syndrome. *Diagnostics.* (2021) 11:849. 10.3390/diagnostics11050849 34065073 PMC8150286

[145] Chen LC Ho TY Shen YP Su YC Li TY Tsai CK et al. Perineural dextrose and corticosteroid injections for ulnar neuropathy at the elbow: a randomized double-blind trial. *Arch Phys Med Rehabil.* (2020) 101:1296–303. 10.1016/j.apmr.2020.03.016 32325164

[146] Lam SKH Reeves KD Cheng AL. Transition from deep regional blocks toward deep nerve hydrodissection in the upper body and torso: method description and results from a retrospective chart review of the analgesic effect of 5% dextrose water as the primary hydrodissection injectate to enhance safety. *Biomed Res Int.* (2017) 2017:7920438. 10.1155/2017/7920438 29226148 PMC5684526

[147] Purita J Lana JFSD Kolber M Rodrigues BL Mosaner T Santos GS et al. Bone marrow-derived products: a classification proposal - bone marrow aspirate, bone marrow aspirate concentrate or hybrid? *World J Stem Cells.* (2020) 12:241–50. 10.4252/wjsc.v12.i4.241 32399133 PMC7202927

[148] Manchikanti L Centeno CJ Atluri S Albers SL Shapiro S Gerard A et al. Bone marrow concentrate therapy in musculoskeletal disorders: evidence-based policy position statement of American Society of Interventional Pain Physicians (ASIPP). *Pain Phys.* (2020) 23:E85–131. 32214287

[149] Pas HI Winters M Haisma HJ Koenis MJ Tol JL Moen MH. Stem cell injections in knee osteoarthritis: a systematic review of the literature. *Br J Sports Med.* (2017) 51:1125–33. 10.1136/bjsports-2016-096793 28258177

[150] Wei P Bao R. Intra-Articular mesenchymal stem cell injection for knee osteoarthritis: mechanisms and clinical evidence. *Int J Mol Sci.* (2023) 24:59. 10.3390/ijms24010059 36613502 PMC9819973

[151] Andia I Martin JI Maffulli N. Platelet-rich plasma and mesenchymal stem cells: exciting, but … are we there yet? *Sports Med Arthrosc Rev.* (2018) 26:59–63. 10.1097/JSA.0000000000000191 29722764

[152] Costa FR Pires L Martins RA Santos M Santos GS Lana JV et al. Orthobiologics revisited: a concise perspective on regenerative orthopedics. *Curr Issues Mol Biol.* (2025) 47:247. 10.3390/cimb47040247 40699646 PMC12025442

[153] D’Souza RS Her YF Hussain N Karri J Schatman ME Calodney AK et al. Evidence-Based clinical practice guidelines on regenerative medicine treatment for chronic pain: a consensus report from a multispecialty working group. *J Pain Res.* (2024) 17:2951–3001. 10.2147/JPR.S480559 39282657 PMC11402349

[154] Dominici M Le Blanc K Mueller I Slaper-Cortenbach I Marini F Krause D et al. Minimal criteria for defining multipotent mesenchymal stromal cells. the international society for cellular therapy position statement. *Cytotherapy.* (2006) 8:315–7. 10.1080/14653240600855905 16923606

[155] Papalia R Zampogna B Russo F Vasta S Campi S Saccone L et al. Adipose-derived stromal vascular fraction processed with different systems for the treatment of knee osteoarthritis: a pilot study on cell proliferation and clinical results. *J Biol Regul Homeost Agents.* (2020) 34:113–9. 33739015

[156] Terrovitis JV Smith RR Marbán E. Assessment and optimization of cell engraftment after transplantation into the heart. *Circ Res.* (2010) 106:479–94. 10.1161/CIRCRESAHA.109.208991 20167944 PMC2826722

[157] Yu S Yu S Liu H Liao N Liu X. Enhancing mesenchymal stem cell survival and homing capability to improve cell engraftment efficacy for liver diseases. *Stem Cell Res Ther.* (2023) 14:235. 10.1186/s13287-023-03476-4 37667383 PMC10478247

[158] Jeyaraman M Bingi SK Muthu S Jeyaraman N Packkyarathinam RP Ranjan R et al. Impact of the process variables on the yield of mesenchymal stromal cells from bone marrow aspirate concentrate. *Bioengineering.* (2022) 9:57. 10.3390/bioengineering9020057 35200410 PMC8869489

[159] Tremolada C Colombo V Ventura C. Adipose tissue and mesenchymal stem cells: state of the art and lipogems^®^ technology development. *Curr Stem Cell Rep.* (2016) 2:304–12. 10.1007/s40778-016-0053-5 27547712 PMC4972861

[160] Doyle EC Wragg NM Wilson SL. Intraarticular injection of bone marrow-derived mesenchymal stem cells enhances regeneration in knee osteoarthritis. *Knee Surg Sports Traumatol Arthrosc.* (2020) 28:3827–42. 10.1007/s00167-020-05859-z 32006075 PMC7669782

[161] Kubrova E Su M Galeano-Garces C Galvan ML Jerez S Dietz AB et al. Differences in cytotoxicity of lidocaine, ropivacaine, and bupivacaine on the viability and metabolic activity of human adipose-derived mesenchymal stem cells. *Am J Phys Med Rehabil.* (2021) 100:82–91. 10.1097/PHM.0000000000001529 32657816 PMC11784493

